# Stepped Evidence Coverage for Plant-Derived Adjuncts in Vulvovaginal Candidiasis: An Evidence Map and a Proposed Mucosal Antifungal Selectivity Window

**DOI:** 10.3390/jof12100722

**Published:** 2026-09-24

**Authors:** Diana Constanța Pelea, Georgiana Ioana Potra Cicalau, Laura Maghiar, Lucian Roman Șipoș, Anca Huniadi, Ruxandra Florina Bodog, Teodora Maria Bodog, Corina Beiușanu, Alicia-Denisa Bereteu-Costa, Csaba Nagy, Alina-Maria Tenche-Constantinescu, Teodor Andrei Maghiar, Mariana Ganea

**Affiliations:** 1 Preclinic Department, Faculty of Medicine and Pharmacy, University of Oradea, Universității Str., No. 1, 410087 Oradea, Romania; diana_pelea@uoradea.ro (D.C.P.); ancahuniadi@gmail.com (A.H.); beiucorina@yahoo.com (C.B.); 2 Department of Dental Medicine, Faculty of Medicine and Pharmacy, University of Oradea, 1 Decembrie Str., 410073 Oradea, Romania; cicalau.georgiana@uoradea.ro (G.I.P.C.); lsipos@uoradea.ro (L.R.Ș.); 3 Department of Psycho-Neurosciences and Rehabilitation, Faculty of Medicine and Pharmacy, University of Oradea, Universității Str., No. 1, 410087 Oradea, Romania; 4 Department of Dermatovenerology, Clinical County Emergency Hospital Bihor, Gheorghe Doja Str., No. 65, 410169 Oradea, Romania; 5 Department of Surgical Disciplines, Faculty of Medicine and Pharmacy, University of Oradea, Universității Str., No. 1, 410087 Oradea, Romania; bodog.ruxandraflorina@student.uoradea.ro (R.F.B.); bodog.teodoramaria@student.uoradea.ro (T.M.B.); 6 Bihor County Emergency Clinical Hospital, Gheorghe Doja Str., No. 65, 410169 Oradea, Romania; 7 Doctoral School of Biomedical Sciences, University of Oradea, Universității Str., No. 1, 410087 Oradea, Romania; costa.aliciadenisa@student.uoradea.ro (A.-D.B.-C.); nagy.csaba@student.uoradea.ro (C.N.); 8 Faculty of Engineering and Applied Technologies, University of Life Sciences “King Mihai I” from Timișoara, 119 Calea Aradului Street, 300645 Timisoara, Romania; alinaconstantinescu@usvt.ro; 9 Department of Surgery, Pelican Hospital, Corneliu Coposu Str., No. 2, 410450 Oradea, Romania; 10 Department of Pharmacy, Faculty of Medicine and Pharmacy, University of Oradea, Nicolae Jiga Str., No. 29, 410028 Oradea, Romania; mganea@uoradea.ro

**Keywords:** *Candida*, vulvovaginal candidiasis, antifungal susceptibility testing, EUCAST, MIC, topical antifungals, local drug exposure, vaginal microbiota, *Lactobacillus*, plant-derived adjuvants

## Abstract

In vulvovaginal candidiasis, agents given almost exclusively by the vaginal route yield minimum inhibitory concentrations that cannot be translated into validated susceptibility categories. We asked whether this deficit is interpretive or empirical, and whether it is measurable. Five reproducible searches were run in PubMed, Web of Science, and Scopus, and a plant-derived subset was extracted from the Web of Science corpus by a predefined term filter. After peer review the filter was tested by dual independent screening (kappa 0.96): it had captured 55% of the eligible records, and the corpus was extended to 321 records. All records were coded independently by two reviewers across five domains, with agreement before reconciliation of kappa 0.75 to 1.00 in the original subset and 0.76 to 0.97 in the added records (0.80 to 0.96 across the whole corpus); prevalence uses the 293 primary studies, and a full-text substudy assessed abstract-level coding. Coverage was stepped: quantitative activity (35.8%) and formulation (36.9%) did not differ, both exceeded biofilm (18.1%) and host-cell (14.0%) testing, and all four exceeded commensal testing (4.4%), a gap of 9.6 to 32.5 percentage points (Cochran’s Q *p* < 0.0001) that the extension widened. Local exposure for topical imidazoles has been quantified in women only in product-registration series of the 1980s and 1990s, varying twentyfold at identical dose. We propose the mucosal antifungal selectivity window, the exposure range in which fungal suppression precedes epithelial injury and commensal disruption, as a hypothesis-generating framework with an operational definition offered for testing; one identified study measured all three domains across overlapping ranges.

## 1. Introduction

Vulvovaginal candidiasis occupies a distinctive position within the spectrum of human fungal disease. It is extremely common, it is frequently self-diagnosed and self-treated, and uncomplicated episodes generally do not require antifungal susceptibility testing [1,2,3]. Recurrent, persistent, and treatment-refractory disease is a different clinical problem: diagnostic uncertainty increases, non-*albicans Candida* species become more relevant, repeated azole exposure may select altered susceptibility phenotypes, biofilm can contribute to therapeutic tolerance, and host and ecological factors become increasingly important [4,5,6,7]. Recurrent VVC alone affects a substantial global population and imposes a durable burden on quality of life [6].

The 2025 global ECMM, ISHAM, and ASM guideline for candidiasis reinforces the need to align microbiological investigation with clinical context rather than performing indiscriminate susceptibility panels [4], while AFST itself has become increasingly standardized: EUCAST uses E.Def 7.4 for broth microdilution of yeasts with breakpoint table version 12.1, valid from 10 April 2026 [8,9], and CLSI maintains an independent framework through M27 and M44, with the fourth edition of M27M44S published in March 2026 [10,11].

This methodological precision exposes a problem that is particularly acute in vaginal disease. Several agents used predominantly or exclusively by local administration, including clotrimazole, miconazole, econazole, fenticonazole, and nystatin, can be evaluated quantitatively in vitro, yet the presence of a measurable MIC does not itself establish a clinically validated topical breakpoint. Likewise, a drug included in a commercial fixed-concentration panel does not automatically acquire an evidence-based S/I/R interpretation.

The central premise of this study is therefore that measurable in vitro activity, clinically interpretable susceptibility, and topical therapeutic efficacy are three different levels of evidence. A standardized MIC is an analytical measurement; a clinical breakpoint is an interpretive construct; local efficacy is a pharmacological and ecological outcome at the vaginal mucosa. This distinction creates a solution-oriented research space rather than a diagnostic void.

The vaginal mucosa adds an ecological constraint that is not captured by conventional systemic AFST frameworks. Its physiological state depends on a protective, usually *Lactobacillus*-dominated bacterial community, and an intervention that eradicates *Candida* while destabilizing that community may be therapeutically counterproductive over the recurrence interval that matters most to patients. Antifungal potency alone is therefore an insufficient development criterion for any locally administered agent, conventional or plant-derived.

The premise that a MIC underdetermines clinical behaviour is not new, and two recent contributions frame it differently from this study. Franconi and Lupetti argued that reference MIC determination should be complemented by fungicidal and tolerance-oriented tests (minimum fungicidal concentration, time-kill analysis, serum fungicidal concentration), directed explicitly at difficult-to-treat invasive disease [12]; mucosal disease lies outside their scope. Jonani argued, for vaginal candidiasis specifically, that the disconnect between phenotypic susceptibility and outcome should be addressed by molecular resistance testing for *ERG11* mutations and efflux regulators [13], resting on a genuine genotype–phenotype disconnect, although the molecular evidence assembled derives substantially from invasive *Candida auris* disease rather than from vulvovaginal isolates, an instance of the pattern documented throughout this study. The three positions share a premise and diverge in what they propose to measure: Franconi and Lupetti would measure more about how the drug kills, Jonani why the organism resists, and this study whether the drug arrives. The third is not reducible to the other two, since an isolate with a wild-type genotype and a low reference MIC will still fail if the achieved local concentration is below the effective range, or will injure the mucosa if it is above the tolerated one. Molecular testing explains why an isolate resists; local exposure and ecological cost explain why a treatment fails.

From this constraint follows the conceptual proposal of this study. We propose the mucosal antifungal selectivity window as a hypothesis-generating development construct for locally administered antifungal and adjunct candidates: the range of local exposure in which fungal suppression or virulence attenuation occurs before epithelial injury and before disruption of protective commensals. The concept is developed in Section 4.8, where it is stated as a theoretical proposal motivated by the evidence gap rather than derived from the mapping data, compared with the pharmacological concepts it extends, and given an operational definition so that it can be tested. It is used here as an organizing principle for evidence appraisal and study design, not as a validated criterion. It is deliberately named for mucosal surfaces in general, because the same three-domain logic should apply to oropharyngeal candidiasis, but every parameter proposed here is defined for the vaginal niche and should not be transferred to the oral cavity without an independent ecological model [14,15]. The same ecological reading is now applied to other niches in which a fungal commensal turns pathogenic, such as *Malassezia* in the hair follicle, where homeostasis, dysbiosis, and disease are described as states of one community rather than as presence or absence of the organism [16].

This study therefore has two components. The first is an evidence-mapping analysis of the literature on plant-derived compounds in VVC, designed to measure where evidence is concentrated and where it is absent, with duplicate coding, formal agreement statistics, and a full-text validation substudy quantifying the limits of abstract-based coding. The second is a translational framework we term the mucosal antifungal selectivity window, proposed in response to those results and to the primary literature on local antifungal exposure. The mapping is the study’s primary result and the only part of it that constitutes evidence. The framework is a concept proposal: its specific form—the three domains, the common exposure axis, the selectivity index, and the exposure tiers—is a theoretical construction that the mapping motivates but cannot validate, and it is offered for testing and amendment rather than for adoption.

The two components answer one question in two steps, and the question should be stated before either is described. It is whether the evidence generated for locally administered antifungal and adjunct candidates in vulvovaginal candidiasis supports the inference routinely drawn from it, namely that demonstrated antifungal activity predicts a useful therapeutic effect at the vaginal mucosa. The analysis of susceptibility testing and of local exposure establishes what a MIC can and cannot support for a topically administered agent, and therefore what an adequate evidence set would have to contain. The evidence map then measures how often that content is present in the plant-derived literature, which is where candidates for this niche are currently generated. Conventional agents and botanical candidates are treated here as two ends of one problem rather than as two subjects: the former have local exposure data and no ecological data; the latter have activity data and neither local exposure data nor ecological data. The map is nevertheless restricted to the plant-derived literature, for two reasons. It is the literature in which new locally administered candidates are currently generated and in which the three-domain question is open, whereas the conventional imidazoles entered use before ecological endpoints were conceived, and their evidence enters this study through the exposure literature of Section 3.3 rather than through the map. And the map deliberately retains botanical studies irrespective of their clinical maturity, because its object is how often the literature tests what it claims; restricting it to agents with clinical evidence would remove the object of measurement. Reading the premise of three evidence levels together with the retention of every plant-derived record is therefore not a contradiction: the domain coding is the operational form of that premise, applied to every study rather than used to select among them.

## 2. Materials and Methods

### 2.1. Design

This study has a two-part design, and the two parts yield different kinds of output. The first is an evidence map: a reproducible retrieval and domain-coding exercise whose results are empirical descriptions of a defined literature corpus. The second is a hypothesis-generating translational framework derived from that map and from the primary literature on local antifungal exposure. The mapping produces the numerical results reported in Section 3; the framework is an interpretive proposal that the mapping motivates but does not validate. No statement belonging to the framework should be read as an empirical finding of the mapping, and no prevalence reported by the mapping should be read as validating the framework. Nor is the framework deduced from the map: the choice of three domains, the shared exposure axis, the selectivity index, and the exposure tiers are theoretical constructions, and Section 4.8 states which of them are new and which extend pharmacological concepts that are long established.

It follows that this is not a systematic review of intervention effects: it does not estimate pooled treatment effects and does not assess risk of bias in individual studies against a validated instrument. It is not a scoping review conducted according to a formal scoping-review methodology, and it is not a bibliometric study, since the coded variable is the presence of an experimental domain rather than a citation or authorship metric. Reporting follows PRISMA-ScR for those items that apply to an evidence map, with the departures stated in Section 5. The study was not prospectively registered. The analysis script and dataset are provided as Supplementary Data S1, and the search strategy, coding manual, and reconciliation record are provided in Table S1.

Throughout, quantitative statements about domain coverage refer to the mapped corpus under the coding strategy defined in Section 2.4, Section 2.5 and Section 2.6. They are not offered as estimates of the vulvovaginal candidiasis literature as a whole, and the wording of the Results and Conclusions preserves that distinction.

### 2.2. Search Strategy and Data Sources

Literature searches were performed in PubMed, Web of Science Core Collection, and Scopus from database inception to 19 August 2026, with all three executed on the same day. No language restriction was applied at retrieval or screening; full-text assessment was conducted in English, with translation where required to determine eligibility. Search strings combined controlled vocabulary and free-text terms across four domains: (i) *Candida* and vulvovaginal candidiasis; (ii) antifungal susceptibility testing, MIC, epidemiological cutoff value, and clinical breakpoint; (iii) biofilm, vaginal microbiota, and *Lactobacillus*; and (iv) plant-derived compounds, essential oils, and named phytochemicals. Reference lists of included articles and relevant reviews were screened manually. EUCAST and CLSI documents were retrieved directly from the issuing organizations and treated as primary methodological authorities. The complete search strategy, including the concept blocks as executed, the translation rules applied between databases, the date of execution, and the record counts at each stage, is provided in Table S1.

### 2.3. Screening and Eligibility

The mapped corpus was not assembled by human screening. It was extracted automatically from the Web of Science population block by a predefined term set applied to the title, abstract, author keyword, and Keywords Plus fields (essential oil, plant extract, phytochem, *plant-derived, botanical*, tea tree, *Melaleuca*, terpinen-4-ol, thymol, carvacrol, curcumin, geraniol, eugenol, terpene*), so the step from 4814 records to the analytic subset involves no inclusion judgement and can be re-executed identically from the exports and scripts in Data S1. The sensitivity of that term set was assessed after peer review by dual independent screening, and the corpus was extended accordingly (Section Sensitivity of the Term Filter and Extension of the Corpus). Single-reviewer judgement entered only the components described at the end of this section and the full-text recoding of Section 2.6, none of which defines the mapped corpus. Two eligibility criteria operated at different levels. For the map, records were retained irrespective of the number of experimental domains subsequently coded, since its object is the composition of the literature rather than the adequacy of any study; prevalence estimates and candidate-level appraisal were restricted to primary experimental and clinical studies, with narrative reviews retained for contextual mapping but excluded from the denominator, and guidelines used as methodological authorities rather than coded. For the narrative synthesis and the candidate-level quantitative appraisal, studies were excluded when the test material was chemically undefined, when the only antifungal evidence lacked a concentration scale, or when the model was not relevant to mucosal exposure; these exclusions did not shape the mapped corpus.

#### Sensitivity of the Term Filter and Extension of the Corpus

Because the term set was constructed around essential-oil and terpenoid chemistry, it was expected to miss plant-derived studies described only by a plant name, a whole-plant extract, a herbal or traditional product, or a named phytochemical, and the reviewers of the first version asked for the screening step to be checked in duplicate. Its sensitivity was therefore tested in three steps, each executed with a retained random-number seed (20260911) by the script screening_sweep_S1.py in Data S1. First, an expanded set of 35 botanical terms (plant and herbal terminology, extract terminology, phenolic classes, and named genera) was applied to the 4639 Article and Review records that the original filter had excluded. Second, the records it flagged were pooled with a random control sample of 150 records flagged by no term, shuffled, and screened independently by two evaluators (L.M. and D.C.P.) blind to stratum and to each other’s decisions, against a single question: whether the record reports, tests, or evaluates a plant-derived preparation or compound in a vulvovaginal candidiasis context, in any study design. Plant material used only as a vehicle or excipient, synthetic derivatives of natural products, marine algae, and studies without a vaginal context were not counted; propolis and honey were recorded in a separate bee-product category and excluded from the plant-derived corpus. Third, a further sweep with named phytochemicals and plant genera was applied to the records still unscreened, and its candidates were screened by the same dual procedure.

Agreement between evaluators was quantified with Cohen’s kappa. Discordant and uncertain records were resolved by discussion and, where necessary, from the full text. Records confirmed by consensus were coded for the five domains by the duplicate procedure of Section 2.5, with the four boundary rules applied prospectively, and appended to the dataset. The sensitivity of the original term set is reported as the proportion of the final corpus it captured, and the residual false-negative rate among records matched by no term is estimated from the random control stratum with an exact binomial interval. The full-text validation substudy (Section 2.6) had been performed on the original 175-record subset and is reported as such.

### 2.4. Evidence Mapping and Domain Definitions

In addition to narrative synthesis, a term-based evidence mapping of the retrieved sets was performed to quantify where evidence is concentrated and where it is absent. Records exported from Web of Science Core Collection on 19 August 2026 were screened for domain-specific terms in title, abstract, and keyword fields, and the resulting counts are reported in the relevant Results and Discussion sections, with their derivation provided in Table S1. These counts describe how often a domain is mentioned, not how often it was experimentally addressed, and are therefore upper bounds; they are used to characterize the distribution of the literature, never as evidence of an experimental result.

### 2.5. Duplicate Coding, Agreement, and Reconciliation

All 175 records in the botanical subset were independently coded by two reviewers against the five domains, working from titles, abstracts, and keywords without access to each other’s coding. The coding manual, including the term sets and the decision rules, is reproduced in Table S1. Agreement was quantified with Cohen’s kappa and was substantial to almost perfect across domains (kappa 0.75 for quantitative antifungal activity, 0.86 for formulation, 0.96 for biofilm, 0.80 for host-cell endpoints, and 1.00 for commensal endpoints). Disagreements were resolved by discussion, and four boundary rules adopted during reconciliation were applied to every affected record: an in vivo dose per unit body weight is not a quantified antifungal concentration; survival or histology in animal models is not host-cell compatibility, which was reserved for measurements on cells or reconstructed tissue; a delivery system counts as a formulation only where intended for vaginal administration; and lactobacilli that form part of the intervention rather than the organism assessed do not count as commensal testing. The rules were adopted after coding rather than specified in advance, which is a limitation; the number of records reclassified under each is given in Table S1. All counts reported below are consensus-coded. The 146 records added after the term-filter check (Section Sensitivity of the Term Filter and Extension of the Corpus) were coded by the same two reviewers under the same manual, with the four rules applied prospectively rather than adopted during reconciliation; agreement for that block is reported separately in Section 3.5.

### 2.6. Full-Text Validation Substudy

Because coding relied on titles, abstracts, and keywords, a validation substudy was performed to estimate how far this underestimates what studies actually report. Forty records were drawn from the 175 by stratified random sampling across four strata defined by the number of domains assigned at abstract level. The strata contained 53, 76, 32, and 14 records for zero, one, two, and three or more domains, from which 10, 12, 10, and 8 records were drawn respectively, oversampling the sparser strata so that multi-domain records were adequately represented. Selection within each stratum used a pseudorandom number generator; the seed was not retained, so the draw cannot be re-executed identically; the record identifiers of the 40 selected are flagged in the dataset supplied as Data S1 (variable validation_sample), so the sample can be verified even though the draw cannot be repeated, and seeds were retained for all sampling performed subsequently (Section Sensitivity of the Term Filter and Extension of the Corpus). The substudy was drawn from the original 175-record subset, before the corpus was extended, and was not repeated on the records added later. The sampled records were recoded from the full text by one reviewer blind to the earlier coding. Full text was obtainable for 29 of the 40 (72.5%); the remainder were inaccessible and are reported as such. The sample contained two narrative reviews, one of which was among the 29 with obtainable full text; it contributed no positive code, so sensitivity estimates are identical whether calculated on all 29 records or on the 28 primary studies among them. Sensitivity of abstract-level coding was calculated against full-text coding, with exact binomial confidence intervals. Because the sampling fractions differed by stratum and the per-stratum samples were small, the substudy is reported as a validation and sensitivity analysis and is not used to extrapolate corrected prevalences to the full set.

### 2.7. Statistical Analysis

Proportions are reported with 95% Wilson confidence intervals. Because the same record can match several domains, differences in domain coverage were assessed with Cochran’s Q for the set of domains jointly, followed by pairwise exact McNemar tests on discordant pairs with Holm adjustment; a chi-squared test treating the domains as independent groups would be inappropriate. Formal temporal-trend analysis was not pursued, because integrated-domain outcomes were too sparse to support informative inference. Inferential analyses of domain coverage were performed within the Web of Science plant-derived subset; prevalence estimates use the 155 primary studies as the denominator. PubMed and Scopus were used for identification and for the cross-database overlap analysis and were not pooled for domain-level inference. Web of Science was chosen as the analytic corpus because its export carries a consistent field structure, including abstracts and both author and indexer keyword fields, so the coding manual could be applied identically to every record; this was a design decision rather than a prespecified hypothesis, and it is noted among the limitations. The analysis script and its output are provided as Supplementary Data S1. The scripts are written in Python 3.13 (Python Software Foundation, Wilmington, DE, USA) and use the pandas (version 3.0), NumPy (version 2.5), and SciPy (version 1.18) libraries. Domain coverage is reported for the original 175-record subset and for the extended corpus; the outcome of interest for the extension was whether the ordering of the five domains was preserved.

A cross-corpus sensitivity analysis was executed according to the protocol prespecified in S1.10. The unmodified term dictionary and coding routine were applied to the plant-derived subsets retrieved independently from Scopus and PubMed, and the prespecified outcome was the ordering of the five domains by prevalence rather than the individual proportions. Because the analysis relies on term matching rather than consensus coding, its results are reported as a check on the ordering only, subject to the overestimation documented in S1.8.

### 2.8. Appraisal of Individual Candidates

Botanical candidates were appraised across seven domains: chemical definition and reproducibility; quantitative antifungal activity; activity against clinically relevant non-albicans or azole-resistant phenotypes; effects on biofilm or virulence; host-cell compatibility; ecological compatibility with vaginal commensals; and formulation or in vivo vaginal progression. Absence of reported toxicity was not treated as evidence of safety, and absence of microbiota data was not interpreted as microbiota preservation. The term microbiota-sparing is reserved throughout for interventions in which preservation of relevant commensals has been directly investigated under exposure conditions relevant to the intended formulation. The scoring rules for the evidence matrix are defined where it is presented and reproduced, with the study-level appraisal, in Table S2. The term sets used for the term-based evidence mapping, and the derivation of each composite count, are given in Table S1.

### 2.9. Use of Artificial Intelligence Tools

Large language models (ChatGPT, GPT-5.6 and GPT-6 models, OpenAI, San Francisco, CA, USA; Claude, Claude Fable 5 and Claude Fable 5.1 models, Anthropic, San Francisco, CA, USA), in the versions available between August and September 2026, were used during the preparation of this manuscript to structure the text, to edit language, to draft and debug the analysis and figure-generation scripts, and to prepare the working files used in the revision, including the screening and coding workbooks of Section Sensitivity of the Term Filter and Extension of the Corpus. All literature searches, screening decisions, domain coding, reconciliation, and interpretation were performed by the authors; no model output was used as a screening or coding decision. The authors reviewed every output and verified every number, statement, and citation against its source, and take full responsibility for the content.

## 3. Results

### 3.1. Retrieval and Cross-Database Yield

The five searches were executed in all three databases on 19 August 2026. The flow of records through retrieval, deduplication, coding, and the two substudies is shown in Figure 1. Record counts, the two invalidated runs, and the cross-database overlap analysis are reported in full in Table S1. Among records carrying a DOI, between 48% and 55% of those retrieved for any single search appeared in only one of the three databases, and no database contained more than approximately two-thirds of the pooled set, so all three were required for identification. Between 11% and 27% of records in each database lacked a DOI and could not be matched across sources; these were treated as distinct, so the proportion unique to a single database is an upper bound. The Web of Science population block is the vulvovaginal candidiasis concept set itself (set #1 in Table S1) rather than the union of the five searches: S1, S2, S3, and S5 each intersect that block with a further concept block and are therefore subsets of it, while S4 was deliberately left unrestricted by disease indexing and lies partly outside it. The block is larger than any individual search for that reason, and it comprised 5385 records, of which 4814 were articles or reviews and formed the denominator for the mapping. The sensitivity of the plant-derived term filter and the resulting extension of the corpus are reported in Section Sensitivity of the Plant-Derived Term Filter and shown as a separate branch of Figure 1.

#### Sensitivity of the Plant-Derived Term Filter

The expanded 35-term sweep flagged 308 of the 4639 excluded records. In the blinded dual screening of these 308 records pooled with 150 random controls (458 records in all), the two evaluators agreed on 97.8% of records, with Cohen’s kappa 0.96 (95% CI 0.93 to 0.98) across the four response categories and 0.97 (0.94 to 0.99) for plant-derived against any other answer. By consensus, 126 of the 308 flagged records and two of the 150 controls were plant-derived studies in a vulvovaginal candidiasis context; 20 concerned propolis, geopropolis, or honey and were set aside as bee products; the 10 discordant records were resolved by discussion and full text to four plant-derived, five not plant-derived, and one bee product, and the seven records marked uncertain by both evaluators to two plant-derived, four not plant-derived, and one unresolved for want of full text. The two positives among the controls were isolated phytochemicals named without any generic botanical term, lawsone and hypericin. A third sweep with named phytochemicals and plant genera flagged 26 of the 4181 records still unscreened, on which the two evaluators agreed completely (kappa 1.00) and confirmed 13, one of which was then excluded at a consistency check because the plant material was the vehicle of a fungal polysaccharide (rule a); a bacterial vaginosis case series of a gel whose vulvovaginal candidiasis counterpart was included remained excluded under rule (e). The original 14-term filter therefore captured 175 of the 321 records of the final corpus (54.5%). It was specific by construction, since every term denotes plant material, but it was built around essential-oil and terpenoid chemistry and missed whole-plant extracts, herbal and traditional preparations, and named phytochemicals, a class dominated by clinical trials of herbal preparations against clotrimazole and by in vitro extract studies. The residual false-negative rate among records matched by no term, two of 150 (1.3%, exact 95% CI 0.2 to 4.7%), implies that roughly seven to 200 plant-derived records may remain unidentified among the 4181 records not screened by hand; that residual is carried as a limitation rather than closed, since closing it would require reading every remaining abstract. The final corpus comprises 321 records (293 primary studies and 28 reviews), and domain coverage is reported below for the original subset and for the extended corpus.

### 3.2. Interpretive Terminology in the Susceptibility Literature

Recent work has begun to quantify MIC distributions for antifungals used in VVC. Kroustali et al. characterized vaginal *Candida* isolates by EUCAST E.Def 7.4 and proposed local epidemiological cutoff values for clotrimazole, miconazole, econazole, itraconazole, fenticonazole, ketoconazole, and boric acid, detecting non-wild-type isolates for azoles in *N. glabratus*, *C. albicans*, and *C. parapsilosis* [17]. The study is important precisely because it creates no therapeutic breakpoint: it shows that topical agents can be studied quantitatively and that wild-type distributions may be epidemiologically informative.

The scale of the interpretive gap can be quantified. Of 567 Web of Science records retrieved by combining vulvovaginal candidiasis with antifungal susceptibility terminology, and restricted to articles and reviews, exactly one mentions epidemiological cutoff values at all: the study cited above. Fifteen mention EUCAST and sixty-six mention CLSI. ECOFF terminology is therefore almost absent from the indexed VVC susceptibility literature, in a field that has generated several hundred susceptibility studies and continues to issue categorical results.

### 3.3. Reported Local Exposure for Topical Agents

This is the point at which the evidence base becomes thinnest, and the deficit has a particular shape. Of 400 records combining vulvovaginal candidiasis with susceptibility terminology and a locally administered agent or dosage form, only 17 matched terminology for any measurement of local exposure, whether in cervicovaginal fluid, vaginal tissue, or by pharmacokinetic analysis, and only four of those concern a topical agent rather than fluconazole. Measurements of this kind do exist, but they belong almost entirely to the product-registration literature of the 1980s and 1990s, and the most informative of them is now four decades old. Houang and Lawrence sampled vaginal fluid and plasma at intervals after a single 300 mg tioconazole ovule in ten women with vaginal candidiasis [18]. Their results establish, in a single small study, most of what this study argues cannot be assumed. A targeted search for pharmacokinetic studies of marketed topical azoles that measured drug in vaginal fluid or tissue in women (PubMed and Google Scholar, 11 September 2026; each agent combined with vaginal fluid, vaginal secretions, cervicovaginal, and pharmacokinetic*) identified no such study published after 2000: the post-2000 pharmacokinetic reports on these products that we found measure plasma concentrations—that is, systemic absorption—rather than local exposure. Within the product-registration literature of the 1980s and 1990s, vaginal-fluid measurements also exist for clotrimazole, where fungicidal concentrations persisted for up to three days after a single 500 mg tablet [19], and for sertaconazole, where levels persisted for over 72 h after a single ovule [20]; the two tioconazole series described below remain the most informative because they report per-woman concentrations at defined sampling times and, in one of them, lactobacillus MICs on the same scale.

Local exposure exceeded systemic exposure by roughly three orders of magnitude: mean plasma concentration was about 21 ng/mL at 8 h and unmeasurable at 24 h in nine of ten women, while mean vaginal concentration at 24 h was about 21 mg/L, still detectable in seven of nine women at 48 h and in two of nine at 72 h. Most consequentially, achieved concentration varied roughly twentyfold between women given an identical dose of an identical product, from 2.4 to 50 mg/L at 24 h, a spread the authors attributed partly to leakage in ambulant patients. Set against the tioconazole inhibitory concentrations for *C. albicans* cited by the authors, 1.6 to 12.5 mg/L, that distribution straddles the inhibitory range: the same treatment placed some women comfortably above it and others at or below it.

This is the empirical basis for treating local exposure as a variable rather than an assumption, since the same isolate and the same product yield different exposures in different women. What was never done is to connect such measurements to anything else: they were generated to characterize a product, not to interpret a MIC, and none paired an individual woman’s achieved concentration with the susceptibility of her own isolate, with mucosal pH, with clinical outcome, or with any effect on the resident microbiota. Where measured concentrations and susceptibility values were placed together at all, it was as a population overlay of MIC90 data from unrelated isolates [21]. The one attempt to characterize a marketed vaginal cream more rigorously did not sample the vagina at all, assessing bioequivalence of two miconazole nitrate creams by dermatopharmacokinetics in forearm stratum corneum on the explicit grounds that repeated vaginal sampling is invasive and lacks a validated method [22]. The quantity on which every argument in this study depends is therefore not unmeasurable, and not unmeasured; it was measured at the target site in a few small product-registration series, the most informative of them four decades ago, and then left unconnected.

A larger series measured the same drug eight years later and shows what happens when achieved exposure is set beside a commensal threshold. Jones et al. determined tioconazole MICs against 32 yeast isolates and 25 *Lactobacillus* strains, and quantified tioconazole in vaginal fluid by bioassay in asymptomatic volunteers after a single 300 mg dose of a 6.5% ointment [21]. The in vitro relationship is favourable, a *Candida* MIC90 of 8 mg/L against a lactobacillus MIC90 of 256 mg/L, a nominal separation of roughly thirtyfold that is wider than any reported for the botanical candidates in Section 4.7, but the delivered exposure appears to have exceeded it: median vaginal fluid concentrations were 884, 424, and 135 mg/L at 24, 48, and 72 h, so the median woman remained above the lactobacillus MIC90 for two days after a single dose. The lactobacilli were unspeciated clinical strains, the yeasts of bloodstream origin, the MICs agar-dilution values of the period, and the fluid values bioassay equivalents rather than chemical concentrations, so the comparison cannot show that lactobacilli were affected in vivo; it shows only that the concentrations recovered lay well above an in vitro lactobacillus value, which is sufficient for the point that a favourable in vitro ratio does not establish that a product operates inside it.

The same literature shows how unstable the exposure denominator is. Within the later series, concentrations at 24 h ranged from 39 to 6207 mg/L among women given an identical dose; between series, the mean concentration at 24 h after 300 mg of tioconazole differs by more than an order of magnitude, about 21 mg/L in one study and about 703 mg/L in the other [18,21]. The earlier study enrolled symptomatic women, whose secretions dilute the applied dose, dosed in the morning with the patients fully ambulant, and its authors suggested night-time administration to improve persistence; the later study dosed volunteers without discharge at bedtime. The determinants of achieved local concentration therefore include the presence of the disease being treated and the time of day of administration, neither of which appears in any susceptibility report nor can be inferred from a product label.

### 3.4. Domain Coverage in the Plant-Derived Subset

Because a review cannot itself test an epithelium or a commensal, prevalence was calculated on primary studies only; the 20 reviews in the subset are reported separately. No review contributed a positive code in any domain, so restricting the denominator changes the proportions and their confidence intervals but not the counts; Cochran’s Q and the pairwise McNemar results are unchanged, because the 20 review records were negative across all five domains.

Among the 155 primary studies, consensus coding identified quantitative antifungal activity in 64 (41.3%, 95% CI 33.8 to 49.2), formulation in 59 (38.1%, 30.8 to 45.9), biofilm in 27 (17.4%, 12.3 to 24.2), a host-cell or epithelial endpoint in 25 (16.1%, 11.2 to 22.7), and a commensal endpoint in eight (5.2%, 2.6 to 9.9). Three studies reported both host-cell and commensal endpoints (1.9%, 0.7 to 5.5). Calculated across all 175 records the corresponding proportions are 36.6%, 33.7%, 15.4%, 14.3%, 4.6%, and 1.7%.

Coverage differed across the five domains (Cochran’s Q = 79.5, df = 4, *p* < 0.0001; Figure 2), and pairwise comparison resolves the difference into three tiers. Quantitative activity and formulation did not differ from each other (Holm-adjusted *p* = 1.0). Both exceeded biofilm, host-cell, and commensal coverage (all Holm-adjusted *p* from 0.0016 to 7.4 × 10^−13^). Biofilm and host-cell coverage did not differ from each other (*p* = 1.0), but both exceeded commensal coverage (*p* = 0.0022 and 0.0045). Within the mapped corpus, coverage therefore has a stepped structure: potency and delivery are coded at one level, biological effect on fungus and host at a second, and effect on the protective community at a third. The tests establish that this ordering is unlikely to be a sampling artefact, but the informative quantity is the size of the gap rather than the *p* value. Commensal testing is lower than host-cell testing by 10.9 percentage points and lower than quantitative antifungal activity by 36.1 percentage points; in absolute counts, eight primary studies reported a commensal endpoint against sixty-four reporting a quantitative antifungal concentration.

#### Cross-Corpus Sensitivity Analysis

The unmodified coding routine and term dictionary were applied to the plant-derived subsets retrieved independently from Scopus (*n* = 316) and PubMed (*n* = 251), following the protocol prespecified in S1.10, with the ordering of domains as the prespecified outcome; denominators are all retrieved records, since term matching does not distinguish document type. In Scopus the ordering was identical to that obtained in Web of Science—quantitative antifungal activity, 41.1%; formulation, 26.9%; biofilm, 18.0%; host-cell, 16.1%; and commensal testing, 13.0%, against 38.9%, 30.9%, 21.7%, 15.4%, and 10.3% by the same routine on the original Web of Science subset—with coverage differing within each corpus (Cochran’s Q 50.7 and 95.3, both *p* < 0.001). Term matching returned commensal testing in 18 of the 175 Web of Science records against eight by consensus coding, reproducing the overestimation factor of about two documented in Section 3.7. PubMed could not be included on comparable terms because its export contained titles only, so the routine had nothing to interrogate in four of its five fields. The full results are given in S1.10.

### 3.5. Agreement Between Reviewers

Before reconciliation, agreement between the two independent coders was substantial to almost perfect: Cohen’s kappa was 0.75 (95% CI 0.65 to 0.85) for quantitative antifungal activity, 0.86 (0.78 to 0.94) for formulation, 0.96 (0.90 to 1.00) for biofilm, 0.80 (0.66 to 0.94) for host-cell endpoints, and 1.00 for commensal endpoints, where the two reviewers agreed on every record. All 42 discordant record-domain pairs were reconciled to consensus, and the four boundary rules adopted during reconciliation are reported in Table S1.

### 3.6. Results of the Full-Text Validation Substudy

The validation substudy shows that abstract-level coding understates the gradient rather than exaggerating it (Figure 3). Across the 29 records recoded from full text, abstract-level coding produced 23 false negatives and no false positives; because record-domain pairs within the same record are not independent, the discordance is reported descriptively, and in 145 pairs it was unidirectional in every instance. Sensitivity was lowest for quantitative antifungal activity (14 of 27, 51.9%, 95% CI 31.9 to 71.3) and host-cell endpoints (five of 10, 50.0%, 18.7 to 81.3), intermediate for formulation (nine of 12, 75.0%) and biofilm (10 of 12, 83.3%), and apparently highest for commensal endpoints, where every full-text positive had been identified from the abstract; with only seven positives the exact lower confidence bound is 59.0%, so this is compatible with a sensitivity substantially below unity. Underestimation was concentrated in the records that appeared sparsest at abstract level: those assigned no domain gained on average one domain on full-text reading, those assigned one gained 1.2, and those already assigned two or more gained less than 0.5.

We do not convert these sensitivities into corrected prevalences. The sample was stratified disproportionately by design, and with six to nine records per stratum a weighted estimator is unstable: applied to abstract-level coding, whose true value across the 175 records is known, it misestimated domain prevalence by up to twelve percentage points in both directions. The substudy is therefore a validation and sensitivity analysis, not a basis for extrapolation.

The asymmetry between domains is nonetheless the informative result, and it does not depend on extrapolation. Abstract-level coding missed evidence in four domains and none in the fifth, the domain on which the central claim rests; given the small number of positives this is weak evidence of high sensitivity rather than proof of it. Within the mapped corpus the ecological domain is therefore not merely the least covered: it is separated from every other domain, including the host-cell domain with which it is often assumed to travel, by a gap that is large in absolute terms and unlikely to be a sampling artefact. Whether the same separation holds in the vulvovaginal candidiasis literature as a whole, retrieved and coded differently, is not established by these data.

### 3.7. The Commensal Subset

Full-text adjudication of the 18 records matching commensal terminology sharpened this picture further. Eighteen matched commensal terminology on automated screening, but consensus coding after independent reading reduced this to eight in which lactobacilli were actually exposed to the test compound and an endpoint measured. The ten excluded records were reviews, practitioner or patient surveys, or studies in which lactobacilli formed part of the intervention rather than the organism whose survival was assessed. Automated term matching therefore overestimated commensal testing by a factor of slightly over two, which is the clearest available measure of how far mention diverges from measurement in this literature. Of the eight, five are primary studies in which a plant-derived preparation was tested against both Candida and lactobacilli under conditions permitting comparison. One further qualifying study was identified in the Scopus set, giving six in total [23,24,25,26,27,28]. Three used species that dominate healthy vaginal communities [24,25,26]; the remaining three used Lactobacillus casei, L. acidophilus, and unspecified lactobacilli. Two tested a vaginal epithelial model alongside the commensal [24,25]. Five reported results against a graded concentration series [23,24,26,27,28]; the sixth applied a single fixed exposure [25].

A graded series is necessary but not sufficient, because the series must cover the concentration at which the compound actually works. The basil essential oil study illustrates the failure mode precisely [26]. Its minimum inhibitory concentration against *C. albicans* was 500 micrograms per millilitre. Commensal testing was generous, covering concentrations up to 2000 micrograms per millilitre with *L. crispatus* viability above 95% throughout, which is a genuine and unusually wide ecological margin. Host-cell testing, however, extended only to 100 micrograms per millilitre, a fifth of the antifungal concentration, and at that upper limit viability of the epithelial-type cell line had already declined to roughly 73%. The compound’s host compatibility at the concentration required to inhibit *Candida* was therefore not tested at all, and the declining trend at the highest concentration examined gives no reassurance that it would have been favourable.

Applying the criteria consistently, the position is starker still. Three of the six tested no host cells at all, reporting *Candida* and commensal endpoints only [23,27,28]. One tested host cells on a range that stopped below the antifungal concentration [26]. One tested all three domains but at a single fixed exposure [25]. Among the primary studies identified under our predefined search and appraisal criteria, one measured *Candida* activity, epithelial compatibility, and commensal compatibility across overlapping concentration ranges [24]. Subject to the retrieval limits stated in Section 2.2 and Section Sensitivity of the Plant-Derived Term Filter, the experimental foundation for microbiota-sparing claims about plant-derived compounds in vulvovaginal candidiasis therefore rested, at the standard this study proposes and within the corpus as originally assembled, on a single paper; the five commensal-positive records added by the extension (Section 3.4) were coded from abstract and keywords, and none of them reports a host-cell endpoint, so none can meet the three-domain standard, and the count is unchanged in the extended corpus.

The most recent addition to this group illustrates how the host domain is displaced rather than measured. Saracino et al. tested five essential oil constituents against 18 *Candida* isolates of vaginal origin and against *L. acidophilus* on the same concentration series, reporting selectivity margins of roughly five- to sevenfold for cinnamaldehyde, α-pinene, and eugenol [28]. No host cell was tested. In place of that measurement, the authors invoked the compounds’ GRAS status and published derived no-effect levels for oral and dermal exposure. Regulatory safety designations for ingestion and skin contact are not evidence of mucosal tolerance at a locally delivered antifungal concentration, and the substitution is worth naming because it is common and because it looks like a safety argument.

### 3.8. Clinical Evidence for Botanical Vaginal Products

Clinical evidence for botanical vaginal products is not absent, and the way it has been generated is instructive. A randomized trial in 100 women compared a polyherbal pessary containing neem extracts, saponins, and mentha-citrata oil with a combination pessary of clotrimazole, tinidazole, and lactobacilli [29]. Conducted at the far end of the developmental sequence proposed here, with none of the intervening stages established, its design carries the cost of that order: women were enrolled on symptomatic discharge rather than confirmed infection, so only 36 had laboratory-confirmed disease and the candidosis subgroups comprised eight and 10 women against a sample size calculated for a 30 per cent difference in symptomatic relief; the primary endpoint was accordingly symptomatic relief (78 and 86 per cent), neither preparation was significantly efficacious against confirmed candidosis, and cure was defined by culture, wet smear, pH, and Nugent score with no quantification of fungal burden, no susceptibility testing, and no measurement of delivered concentration.

Two features bear on the framework. No ecological endpoint was recorded: lactobacilli were assessed only as a component of the bacterial vaginosis criteria, and the comparator itself contained lactobacilli, so a trial of a broad-spectrum botanical preparation administered into the vagina generated no data on the community it might disturb. And the only host tolerance signal is vaginal burning in 16 per cent of women receiving the polyherbal pessary against 4 per cent receiving the comparator, an epithelial signal recorded as an adverse event in treated women rather than measured in a preclinical model, the sequence this study argues should be reversed; a mint oil is among the constituents, and the one study to test a *Mentha* oil against *Candida* and lactobacilli on a shared scale found the commensals more susceptible [27]. This trial was not captured by the original 14-term filter and entered the corpus through the extension of Section Sensitivity of the Plant-Derived Term Filter, which illustrates the class of study that filter missed.

## 4. Discussion

### 4.1. Principal Findings

Three results define what follows. Within the mapped corpus, domain coverage in the plant-derived literature is stepped rather than continuous, with commensal testing separated from every other domain, including host-cell testing, by 10.9 to 36.1 percentage points; re-applying the same routine to an independently retrieved Scopus corpus preserved that ordering, as prespecified in S1.10. In the validation sample, abstract-level coding missed evidence in four domains and none in the commensal domain; with only seven commensal-positive records and a lower confidence bound of 59%, this is weak evidence that the ecological deficit is not an artefact of shallow reading, and it is not repeated below as if it were established. Extending the corpus after the term-filter check preserved the ordering and widened the gap (Section 3.4). And local exposure, the quantity on which any concentration-referenced criterion depends, has been measured in women for conventional vaginal imidazoles only in a few small product-registration series of the 1980s and 1990s, the two most informative of them four decades old, and has never been connected to the susceptibility of the isolate or to outcome.

### 4.2. What a Quantitative MIC Supports and What It Does Not

The principal strength of reference broth microdilution is reproducibility. EUCAST E.Def 7.4 and CLSI M27 define controlled experimental conditions under which a quantitative MIC can be generated [8,10]. Standardization of inoculum, medium, incubation, and endpoint reading is what makes a MIC comparable between laboratories; it is not what makes it clinically predictive [30].

Even a validated category predicts outcome loosely. A substantial minority of infections caused by isolates categorized as resistant nevertheless respond to therapy, so categorical agreement with outcome is partial rather than deterministic [12]. A breakpoint is a probabilistic instrument even where fully validated, and treating it as deterministic is a misreading that predates the topical problem addressed here.

Clinical interpretation requires an additional evidentiary layer. EUCAST maintains clinical breakpoints and epidemiological cutoff values as distinct resources and documents the pharmacokinetic, pharmacodynamic, and clinical reasoning behind each breakpoint in rationale documents [9,31]; CLSI similarly separates breakpoints from epidemiological cutoff values [11]; and where no breakpoint exists, both organizations state how a MIC may and may not be reported [32,33]. For VVC the distinction matters because local exposure differs markedly from systemic pharmacokinetics: vaginal dosage forms can transiently produce concentrations far above plasma levels, but the biologically relevant concentration changes with formulation release, dilution, mucus and tissue binding, and mechanical clearance, so a breakpoint derived from systemic exposure cannot be repurposed as a topical one, and the direction of the error is unpredictable.

The vaginal environment shows the same point. VVC occurs in an acidic environment while reference AFST is performed at near-neutral pH, and lowering test pH alters MICs for multiple antifungals, substantially for azoles and particularly for *Nakaseomyces glabratus* [34]. This does not justify changing the reference method, which would lose reproducibility without gaining validated interpretation. It justifies a separate translational layer in which vaginally relevant conditions are studied explicitly and results described as mucosal-model activity rather than as a second clinical MIC.

### 4.3. Interpretive Practice in the Laboratory

The terms MIC, ECOFF, and clinical breakpoint should remain terminologically strict. A MIC describes a measured endpoint; an ECOFF separates the wild-type population from isolates with unusual susceptibility distributions; a clinical breakpoint supports a treatment-oriented category and requires pharmacokinetic, pharmacodynamic, and outcome evidence. An isolate outside a wild-type distribution should therefore not be labelled clinically resistant to a vaginal preparation, a low experimental MIC is not proof of local efficacy, and a comparatively high topical MIC does not establish that a formulation delivering a hundredfold higher local concentration will fail.

Three consequences follow for practice. First, the appropriate response to the absence of a topical breakpoint is not to preserve pseudo-S/R categories inherited from older commercial panels. Second, laboratories generating topical MICs should report them as quantitative activity with an explicit statement that no validated interpretive criteria exist, in line with the general principle applied to organism and drug combinations without breakpoints [32,33]. Third, the gap should be closed by evidence rather than by convention: better quantitative distributions, characterization of reproducibility across laboratories, measurement of local drug exposure, and prospective linkage of MIC to treatment outcome. Only such data can establish whether a topical MIC has clinically useful predictive value. This analytical to translational discontinuity is summarized in Figure 4A. The laboratory practice that follows from it—when testing should be escalated, how fixed-concentration commercial panels differ from quantitative MIC systems, and a three-tier reporting vocabulary—is set out in Supplementary Note S3 with Tables S3 and S4; only the points that bear on the framework are kept here.

One reason the gap has persisted without being felt was demonstrated by a regulatory methods study. Two miconazole nitrate vaginal creams, 2% differing in vehicle, were compared by two routes: dermatopharmacokinetic assessment found them not bioequivalent, with test to reference ratios of 0.666 and 0.523 and confidence intervals well below the acceptance range, while a clinical trial in 106 women with culture-positive vaginitis found the same products bioequivalent, with clinical cure of 92 and 87 per cent and mycological cure of 96 per cent for both [22]. The authors’ explanation is the part that matters: the antifungal response saturates, so bioequivalence can be attained by delivering drug in excess of what the target requires, and a clinical endpoint recorded at the plateau cannot resolve differences below that excess.

Two consequences follow. First, clinical cure cannot be treated as evidence that local exposure is adequate or even known, since it proved insensitive to a nearly twofold difference in delivered drug; the weak relationship between MIC and outcome documented for antifungal therapy in general [12] therefore has a mundane component in the topical setting, namely that outcomes are recorded at the top of the dose-response curve. Second, the excess that secures the clinical result is not biologically free: drug delivered beyond what is needed to suppress *Candida* still reaches the epithelium and the resident microbiota, where the response has not saturated. The ureasil–polyether ovule data in Section 4.7.3 make this concrete for the same agent, with about 135 mg/L released in vitro against an epithelial effect concentration near 6 mg/L [24]. Efficacy insensitive to overdelivery together with epithelial injury sensitive to it is the configuration in which a selectivity window is worth defining, and in which its absence is least likely to be noticed.

Two points from that practice bear directly on the argument. Species-level identification should precede any expansion of the antifungal panel, because *Candida albicans*, *Nakaseomyces glabratus*, *Pichia kudriavzevii*, *Candida tropicalis*, and the *Candida parapsilosis* complex differ in baseline susceptibility, intrinsic resistance, and mechanisms of acquired resistance [35,36], and because name changes of this kind are themselves a source of interpretive error unless a stable clinical nomenclature is followed [37]. And treatment failure should not be equated with microbiological resistance: adherence, duration, formulation-related exposure, diagnostic error, biofilm, host factors, reinfection, and ecological disturbance can all produce persistent symptoms in the presence of a fully susceptible isolate, which is why indiscriminate panels generate false reassurance in one direction and unwarranted escalation in the other.

Commercial systems show why analytical output must be kept apart from clinical interpretation. Quantitative colorimetric microdilution systems are method dependent, with essential agreement of at least 90% achieved only for selected agents and systematic differences in MIC distributions between systems [38], and EUCAST now requires in-house qualification of a commercial test before its breakpoints are adopted [39]. Fixed-concentration panels that include several local agents have shown poor performance against reference microdilution [40] and are still used for descriptive profiling [41]; in a series of 300 genital yeast isolates in which every strain was screened by the panel and 43 flagged isolates were re-tested by broth microdilution [42], reduced fluconazole susceptibility was confirmed in 12 per cent of the 150 *C. albicans* isolates and 4 per cent of the non-albicans isolates when expressed against all isolates examined, yet every confirmed isolate came from the 43 flagged by the screen, so the confirmed *C. albicans* alone account for at least 18 of those 43. Both denominators are legitimate; the distance between the two counts is the point: screen-then-confirm supports statements about how often a flagged phenotype is confirmed, not about the MIC distribution of a population, which is what the surveillance role proposed here requires.

The corresponding laboratory vocabulary—three reporting levels with their permitted conclusions and the conclusions to avoid—is given in Table S3; it requires no new susceptibility category and no change to accredited methods, and changes only what the laboratory is willing to assert.

### 4.4. Conventional Topical Agents as Comparators

Clotrimazole, miconazole, econazole, and fenticonazole remain clinically established local antifungals, but widespread use does not generate MIC breakpoints for vaginal treatment; their research value lies in species-specific MIC distributions, detection of unusual phenotypes, and prospective correlation with outcome, and a fluconazole result should not be used to withhold a topical imidazole, since cross-azole associations in vaginal isolates do not establish cross-resistance [17,35] and fluconazole is a poor proxy for a topical imidazole even in vitro [21]. Where no validated criterion exists the vacuum is filled informally: in one series of genital yeast isolates, categorical resistance to clotrimazole was assigned above a MIC of 1 mg/L on the authority of an earlier publication and nystatin was read against a limit suggested by other investigators, with different MIC endpoints for the two agents [42]; the thresholds are explicitly attributed, and the point is the mechanism, not the study. The imbalance in how these agents have been studied is itself informative: within the 567 susceptibility records, fluconazole is mentioned in 347, clotrimazole in 85, nystatin in 71, and miconazole in 64, but econazole is mentioned in seven and fenticonazole in five, so local MIC distributions for several established vaginal imidazoles remain unavailable rather than merely uninterpreted. Fluconazole should remain the principal standardized comparator wherever an applicable breakpoint exists, and nystatin a mechanistically distinct one; the analytical value and interpretive limits of each agent, and the comparator argument in full, are given in Supplementary Note S3 and Table S4.

### 4.5. Biofilm and the Vaginal Microenvironment

Biofilm provides a biologically plausible explanation for part of the discordance between planktonic MIC and therapeutic response. *Candida albicans* biofilms contain an extracellular matrix, metabolically heterogeneous subpopulations, altered stress responses, and cells with markedly increased tolerance to conventional antifungals [43,44]. Matrix components contribute directly to antifungal tolerance by limiting effective drug exposure at the cell surface rather than by altering the drug target [45]. A candidate intended for recurrent or refractory VVC should therefore not advance on the basis of planktonic MIC alone. Cell-wall remodelling under host and drug pressure, with reorganization of the β-glucan, chitin, and mannoprotein layers, provides the mechanistic background for why matrix components limit drug access and enable immune evasion [46].

Biofilm testing should distinguish prevention of formation from activity against mature biofilm, report biomass, metabolic activity, and viable burden separately, and include sub-MIC concentrations, since compounds may suppress adhesion, filamentation, or matrix formation well below growth-inhibitory concentrations. In the vagina, moreover, biofilm cannot be modelled as a monomicrobial structure: recurrent VVC has been described as a dynamic interkingdom process in which *Candida* and *Lactobacillus* populations interact within the same biofilm [47], so an anti-biofilm effect in a pure *C. albicans* biofilm characterizes the compound, not the disease, and an agent that disperses fungal biofilm while removing the commensal biofilm may leave the niche more, not less, susceptible to recolonization.

The extent to which the field has not done this can be measured. Among 1125 Web of Science records combining VVC with biofilm, microbiota, or epithelial terminology, 383 matched biofilm terminology but only 25 also matched polymicrobial, mixed-species, interkingdom, dual-species, or co-culture terms. Term matching cannot establish what proportion of the remainder used monomicrobial designs; it establishes only that the design is rarely signalled.

This is particularly important for plant-derived adjuncts. A compound with a moderate MIC but strong anti-virulence activity at concentrations well tolerated by epithelium and commensals may be more useful than a highly fungicidal but non-selective compound. That comparison cannot be made from MIC tables alone; it requires the three domains to be measured on a common concentration scale, which is the argument developed in Section 4.8.

### 4.6. The Protective Microbiota as a Development Constraint

The evidence base for locally administered antifungals and adjuncts is markedly asymmetric: anti-Candida activity is abundant, host safety evidence is less common, and direct microbiota preservation evidence is sparse. This asymmetry is not a minor reporting gap. It is the central methodological weakness of the field, because the healthy vaginal niche is strongly structured by its bacterial community rather than simply by the absence of fungi. Community composition in the lower reproductive tract is increasingly treated as a clinical variable in its own right [48], which makes its near-absence from the development evidence for locally administered antifungals the more conspicuous.

*Lactobacillus crispatus* can inhibit *C. albicans* growth and hyphal formation and modulate the expression of virulence-associated genes [49,50], modulate epithelial defence through innate immune signalling [51], and contribute to an anti-*Candida* ecological state through biofilm-associated mechanisms [52]. Anti-adhesion activity has also been characterized for defined *L. crispatus* strains under controlled cultivation conditions, which is directly relevant to any product intended to preserve or restore colonization resistance [53]. *L. gasseri* and *L. jensenii* are further relevant components of healthy vaginal communities, while *L. iners* requires separate interpretation because of its distinctive ecological behaviour and its association with less stable community states [48]. Two recent studies show what the field is being asked for. A lipopeptide biosurfactant from vaginal *L. gasseri* prevented and dispersed *Candida* biofilm, was non-cytotoxic to HeLa cells, and left the taxonomic composition of a bacterial microcosm essentially unchanged, an anti-*Candida* agent characterized explicitly for ecological compatibility rather than for potency alone [54]; and genome-scale metabolic modelling of 159 vaginal *Lactobacillus* strains, with metagenomic and in vitro confirmation, identified specific strains and metabolites, among them lactate, formate, and malate, as inhibitors of *C. albicans* [55]. Both treat the commensal as the reference organism rather than as an afterthought, which is the orientation the framework requires.

Which organisms the field actually studies is not aligned with which organisms dominate the healthy vagina. Within the same 1125 records, 428 mention lactobacilli, but the single most frequently named species is *Lacticaseibacillus rhamnosus*, in 96 records, ahead of *L. crispatus* in 69; *L. gasseri* appears in 29, *L. jensenii* in 16, and *L. iners* in 20. Much of the ecological literature on VVC is therefore built on probiotic strains selected for gastrointestinal or manufacturing properties rather than on the species that define vaginal community state types, and the species whose behaviour most complicates the interpretation of a healthy community, *L. iners*, is among the least studied.

Which commensal is chosen also determines the answer. In the tea tree oil suppository data discussed in Section 4.7.1, the selectivity margin over lactobacilli was roughly eightfold and over *Bifidobacterium animalis* twofold, for the same product on the same axis [23]. A microbiota-sparing claim is therefore meaningless without a stated panel justified by current vaginal ecology. Saracino et al. selected *L. acidophilus* on the stated grounds that it constitutes some 90 per cent of the vaginal Doderlein complex [28]; community state type data do not support that description, the dominant taxa being *L. crispatus*, *L. iners*, *L. gasseri*, and *L. jensenii* [48]. A margin measured against it is a real measurement against the wrong organism.

Nor can selectivity be assumed to follow from chemical class or botanical family. Iseppi et al. tested two Lamiaceae oils against the same vaginal *C. albicans* and *Lactobacillus* isolates under identical conditions [27]. For *Lavandula x intermedia*, *Candida* was inhibited at 9 to 18 micrograms per millilitre and lactobacilli at 72 to 144; for *Mentha arvensis* the ordering reversed: two oils, one family, one laboratory, one assay, and opposite verdicts. Reasoning from constituent chemistry would not have predicted which was which; only measuring both organisms on the same scale reveals it.

It should also not be assumed that the microbiota is the constraint that binds first. For miconazole in the ureasil–polyether system, vaginal epithelial cells were affected at roughly a sixteenth of the concentration that affected *L. jensenii* [24]. Ecological compatibility and host compatibility are separate requirements, and which of the two is limiting is a property of the compound, not a general rule. A programme that assesses only one of them cannot know which it has missed.

A vaginal candidate should therefore be tested against at least *L. crispatus*, *L. gasseri*, and *L. jensenii*, under the same concentration series and exposure duration used for fungal testing, in organism-appropriate media with matched vehicle controls, with ratios derived across non-bridged assays reported as exploratory. What cannot be relaxed is the concentration axis: a commensal tested at a single concentration, or over a range excluding the antifungally active concentration, yields a number that cannot support any selectivity claim. Advanced models should progress to mixed communities, and there is direct evidence that the simpler design misleads: in Fernandes et al., *L. gasseri* was unaffected during co-colonization with *C. albicans* but more susceptible when exposed alone [25]. Ecological compatibility is a property of the community, not of the organism in isolation.

The three domains are also rarely integrated. Of the 1125 records, 428 address microbiota, 383 biofilm, and 447 epithelial endpoints, yet only 25 touch all three, and only six combine commensal and epithelial endpoints with an explicit concentration scale. The literatures exist and are individually substantial; what is missing is the shared axis on which a selectivity judgement could be made.

A compound should only be described as microbiota-sparing when this property has been directly demonstrated. Lack of ecological data should be stated as an evidence gap, not interpreted as safety. Applying this rule strictly reclassifies most of the current botanical literature, including several compounds with otherwise excellent antifungal credentials.

### 4.7. Priority Botanical Candidates

The botanical antifungal literature is extensive but translationally uneven: many essential oils, extracts, and phytochemicals inhibit *Candida* in vitro and many have anti-biofilm activity [56], but potency alone is a weak basis for vaginal development, natural origin does not guarantee epithelial safety, and broad antimicrobial action is undesirable in an ecosystem that depends on protective commensals. A prior constraint is chemical definition: a preparation is testable to the standard proposed here only if its composition is defined and reproducible, which analytical profiling of standardized extracts makes achievable [57], and an unstandardized oil identified only by its plant of origin cannot be placed on a concentration axis shared with an epithelial or commensal endpoint.

The most defensible therapeutic role for botanical products is therefore adjunctive rather than substitutive. A standardized plant-derived compound may potentiate an established antifungal, reduce adhesion, inhibit the yeast to hypha transition, weaken biofilm, modify local drug distribution, or permit a lower conventional antifungal exposure for the same effect. This reframing replaces the scientifically weak question of whether a plant extract can replace an antifungal with a tractable one: whether it adds selective value to an evidence-based local regimen. The comparative evidence position of the candidates discussed below is set out in Table 1.

#### 4.7.1. Tea Tree Oil and Terpinen-4-ol

Tea tree oil from *Melaleuca alternifolia* provides one of the most informative developmental trajectories: quantitative activity against *C. albicans* and non-albicans *Candida*, including isolates with reduced azole susceptibility, with the constituents responsible identified [58,59], and terpinen-4-ol, a defined constituent, active against azole-susceptible and azole-resistant isolates and efficacious in an experimental vaginal candidiasis model [59]. The progression from a chemically variable oil to a defined constituent with in vivo vaginal data is the sequence most botanical candidates never complete.

The 2015 vaginal suppository study by Di Vito et al. evaluated *Candida* and selected beneficial bacteria within one experimental framework, on a single shared concentration axis [23]. Tea tree oil inhibited *C. albicans*, including azole-resistant isolates, at 0.25% *v*/*v*, with fungicidal concentrations of 0.25 to 0.5%, whereas *Lactobacillus* strains required 2 to 4% and *Bifidobacterium animalis* only 0.5%. Under the convention of Section 4.8.1, the microbiota selectivity ratio pairs the lowest disruptive concentration in the protected domain with the lowest effective concentration in the fungal domain: 2% against 0.25%, an eightfold margin against the most susceptible lactobacillus. The sixteenfold figure sometimes derived from the same data pairs the least susceptible lactobacillus (4%) with the lowest fungicidal concentration and is the upper bound of the full cross of the two ranges (fourfold to sixteenfold); it is not the value the framework would report. Against *B. animalis* the same computation gives twofold, so the window is wide or narrow depending on which commensal constrains it. The marketed suppository contained 0.5% *v*/*v*, fungicidal for *Candida*, sparing for lactobacilli, and precisely at the concentration inhibiting *B. animalis*. The authors reasoned in these terms, identifying 1% as a defensible upper bound.

This is a selectivity window reported a decade before the term proposed here, and it shows why the concept needs a stated commensal panel: the same product is selective or borderline depending on which organism is measured. Fungicidal activity was retained at a pH of 5 as well as a pH of 7, an advantage no MIC at reference pH would reveal [34]. It does not prove preservation of the vaginal microbiome, since the bacterial panel did not represent the dominant community state types, and cytotoxicity data for the oil and its components come from separate epithelial models [60]. Tea tree oil is a benchmark for how to test, not a validated microbiota-sparing therapy.

#### 4.7.2. Thymol

Thymol has well-documented activity against *C. albicans* biofilm formation and mature biofilm [61], and terpenes including thymol, carvacrol, and geraniol inhibit established *Candida* biofilms [62], but its translational value lies in combination pharmacology: in biopolymer vaginal films for resistant vaginal candidiasis, thymol increased fluconazole distribution within vaginal tissue and the combination improved anti-*Candida* activity [63], which is the type of evidence an adjunct paradigm requires, with the phytochemical modifying the local performance of the conventional drug rather than competing with it on MIC. The unresolved issue is ecological selectivity: thymol has broad antimicrobial activity, so microbiota-sparing claims for thymol-containing products are premature until the antifungal range is compared directly with the concentrations affecting *Lactobacillus* spp. and vaginal epithelial cells within the intended formulation.

#### 4.7.3. Curcumin

Curcumin illustrates why a free-compound MIC can misrepresent translational potential in both directions: poor aqueous solubility makes formulation central to local delivery, so an unfavourable MIC may reflect the vehicle rather than the molecule, and although experimental vaginal candidiasis work supports biological activity [64], the relevant development strategy is controlled co-delivery.

Bezerra et al. developed hybrid vaginal ovules for co-delivery of curcumin and miconazole against *C. albicans*, with synergy allowing both agents to act at concentrations several-fold lower than alone [65], and subsequent work with a ureasil–poly(propylene oxide) matrix extended the system to non-albicans strains and, uniquely in this corpus, measured antifungal activity, epithelial compatibility, commensal compatibility, and in vitro release from the finished dosage form within one study [24]. The numbers are set out in full because they overturn an assumption that runs through the microbiota-sparing literature, and because they are the one dataset on which the selectivity index of Section 4.8.1 can be computed.

Miconazole inhibited *C. tropicalis* at 1.25, *C. parapsilosis* at 2.5, and *C. glabrata* at 12.5 micrograms per millilitre. Viability of *L. jensenii* fell significantly above 95 micrograms per millilitre. Viability of VK2/E6E7 vaginal epithelial cells, however, fell below 40% at 5.94 micrograms per millilitre. Curcumin was markedly better tolerated on both counts, without effect on the commensal at the highest concentration tested and with only modest epithelial impact above 64 micrograms per millilitre, and the unloaded matrix affected neither. Approximately 135 micrograms per millilitre of miconazole was released from the ovules within 24 h.

Three conclusions follow, and none is comfortable. First, for miconazole the binding constraint appears to be the epithelium rather than the microbiota: marked epithelial cytotoxicity occurred roughly sixteenfold below the concentration reducing *L. jensenii* viability, and although neither value is a validated threshold and the two derive from different assays, a programme testing only lactobacilli would have inferred a wide margin where the data suggest none. Second, against *C. glabrata* the inhibitory concentration exceeds the epithelial effect concentration outright, so on these measurements there is no window for that species. Third, the concentration released in vitro exceeded both effect concentrations, and whether the achieved mucosal concentration does so is the quantity that has not been measured; by lowering the miconazole concentration required, curcumin moves the necessary exposure toward the epithelial effect concentration rather than past it, which is where the adjunct rationale acquires quantitative meaning.

One caveat is essential: a submerged monolayer of an immortalized cell line may overestimate cytotoxicity relative to an intact stratified epithelium bathed in cervicovaginal fluid, so the absolute epithelial effect concentration is not a clinical limit. The relative ordering is the finding: for this agent, epithelial sensitivity precedes ecological disruption, and any framework that measures only the latter will overestimate the available margin.

#### 4.7.4. Geraniol

Geraniol provides a complementary, anti-virulence rationale and the thinnest evidence base discussed here. In a murine vaginal candidiasis model, intravaginal geranium oil and geraniol reduced fungal burden and local inflammation when administered together with vaginal washing, and low concentrations interfered with mycelial growth [66]; the dependence on a mechanical clearance step means the effect should be reproduced without washing before it is attributed to the compound. Terpenes including geraniol also inhibit established *Candida* biofilms [62]. Because *Candida* is frequently a commensal rather than an invariable pathogen in the vagina, control of pathogenic behaviour and restoration of mucosal homeostasis, rather than eradication of the genus, is a legitimate therapeutic goal, and an anti-virulence candidate requires different primary endpoints from a fungicidal one: filamentation, adhesion, matrix formation, and host inflammatory response, with MIC as a secondary descriptor. That such candidates accumulate few publications regardless of merit is itself a consequence of the endpoints the field favours.

### 4.8. The Mucosal Antifungal Selectivity Window

The three preceding sections describe three separate literatures that rarely share a concentration axis: on the counts reported above, fewer than one per cent of the retrieved VVC records place commensal and epithelial endpoints on an explicit concentration scale together. The proposal of this study is that they must.

We define the mucosal antifungal selectivity window as the range of local exposure within which a candidate achieves fungal suppression or virulence attenuation, while epithelial integrity and protective commensal function are preserved. It has three components, each measured against the same concentration series and exposure duration, under vaginally relevant conditions harmonized as far as the biology of each system permits: *Candida* activity, epithelial compatibility, and commensal compatibility. The window is the exposure interval in which the first is achieved and the other two are not yet compromised. Once the framework is operationalized, a candidate would progress only when the local exposure achievable with the intended formulation falls inside that interval.

The gap is unlikely to be an artefact of indexing: a deliberately broad search without a vulvovaginal population block retrieved 1208 Web of Science records, of which only 13 combine an explicit concentration scale with both commensal and host-cell endpoints and only two concern the vagina, so broadening retrieval increases yield roughly sevenfold while adding almost nothing that meets the selectivity criterion.

One recent study implements the three domains and states the principle explicitly: Fernandes et al. evaluated the vapour phase of white thyme essential oil against *C. albicans* biofilm and against *Lactobacillus gasseri* during single and co-colonization of a reconstituted human vaginal epithelium, with epithelial damage assessed by lactate dehydrogenase release, and framed commensal preservation as a design requirement [25]. What it does not establish is a window: the oil was applied as a single fixed exposure and fungal susceptibility characterized by inhibition zones rather than a concentration series, so selectivity is demonstrated at one point rather than across a gradient, which answers whether a preparation can be selective but not at what exposure it ceases to be.

What is novel here requires stating precisely, and the concept has antecedents that the first version of this manuscript did not cite. The selectivity index, the ratio of a host toxic concentration to an effective one, typically CC50 to IC50, is a standard screening quantity in antiviral, antiparasitic, and natural-product pharmacology, where parallel cytotoxicity testing over an adequately broad dose range is a stated requirement before a natural product is taken further [67]; the therapeutic index expresses the same relationship at the level of exposure in humans [68]; and antifungal pharmacokinetic–pharmacodynamic indices relate drug exposure to the MIC for systemic therapy [69]. Both Di Vito et al. and Fernandes et al. produced selectivity relationships in this niche without naming them [23,25]. Relative to those concepts, the window proposed here changes three things and nothing else. It adds a third domain, so that the upper bound is the smaller of an epithelial and a commensal threshold rather than a single cytotoxicity value, and the window is defined by the more restrictive protected domain. It requires the three concentrations to lie on one declared exposure tier, formulation-specific rather than nominal, because the local exposure of a vaginal product is neither a plasma concentration nor an applied one. And it specifies which endpoints, species panel, and reporting elements are admissible (Table 2), so that two laboratories can compute the same quantity and an untested domain returns an undefined result rather than a favourable ratio. The three-domain extension and exposure-tier requirement are the contribution; the ratio itself is a relabelling of a known concept, and it is presented as such.

Two datasets in this corpus already contain most of what the definition requires. Di Vito et al. determined tea tree oil concentrations inhibiting *Candida*, lactobacilli, and bifidobacteria on one axis, and the resulting ratios differ by nearly an order of magnitude depending on which commensal is chosen [23]: the window is defined by the most susceptible protected organism, not the most convenient. In the ureasil–polyether ovule system, fungal inhibitory concentrations, experimental epithelial and commensal effect concentrations, and the concentration released from the finished dosage form were measured on one axis [24,65], describing a window bounded below by fungal inhibition, above by epithelial injury well beneath the commensal effect concentration, absent for one *Candida* species, and exceeded by the in vitro release concentration; the missing quantity is the achieved mucosal concentration. The concept exists not to confirm that a candidate is selective, but to reveal where it is not.

Before the framework can function as an applied criterion rather than an organizing principle, several parameters must be fixed by convention: the fungal endpoint and its magnitude, the epithelial readout and threshold, the commensal endpoint and species panel, and the exposure duration, pH, matrix, and concentration basis on which all three are determined. A first specification is proposed in Section 4.8.1 and Table 2, offered for testing and amendment rather than for adoption. Until those conventions are agreed, the window should be read as a framework for organizing evidence and designing studies, not as a criterion ready to be applied to a candidate.

#### 4.8.1. An Operational Definition

A construct without a computational definition cannot be tested, so the conventions are specified here. Table 2 gives, for each domain, a primary metric, admissible alternatives, and the reporting requirements without which a value is not interpretable; it is offered as a proposal for consensus, not a validated standard, so that two laboratories can compute the same quantity from the same data. Applied to the six studies of the commensal subset in S2.5, the index proves computable in one, closed in one, and undefined in four.

Let CF denote the fungal effective concentration, the lowest concentration at which the stated fungal endpoint is met; CE the epithelial threshold concentration, the lowest concentration at which the stated epithelial injury threshold is crossed; and CM the commensal threshold concentration, the lowest concentration at which the stated commensal endpoint is crossed for the most susceptible species in the declared panel. All three endpoints should be determined over a harmonized concentration series and matched exposure duration, using model-appropriate media under vaginally relevant conditions, and at the same declared exposure tier (Section 4.8.2). Where matrix or pH cannot be identical, bridging controls should be used and cross-assay selectivity ratios should be regarded as exploratory.

The window is the interval bounded below by CF and above by the smaller of CE and CM, and its width is the dimensionless selectivity index S, the ratio of that smaller upper bound to CF. Where S exceeds 1 the window is open, and the bounding ratios CE/CF and CM/CF identify which domain constrains it. Where S is 1 or less the window is closed: the candidate reaches epithelial injury or commensal disruption at or below the concentration required for antifungal effect. A closed result is conditional on the compound, formulation, and conditions tested, since reformulation or combination may lower the exposure required, in which case the three concentrations are redetermined and the index recomputed (Section 4.9 and Section 4.10); it is reported as closed, and never as a negative value, since the interval is empty rather than inverted. The intermediate case, S above 1 for one protected domain and not for the other, is the configuration of both the basil dataset in Section 3.7 and the ureasil–polyether dataset in Section 4.7.3, and is what a single summary ratio conceals; the window is therefore defined by the more restrictive protected domain, and never by the more convenient domain.

The sequence by which the index is obtained, and the two outcomes that return an undefined result rather than a number, are shown in Figure S1. Two further rules are required for the index to behave consistently. First, an untested domain is not a satisfied domain: where CE or CM has not been measured, S is undefined and must be reported as undefined rather than computed from the domains that were measured. Second, the tested range must span CF. Where the highest concentration examined in a protected domain lies below CF, that domain has not been tested at the concentration that matters, and the correct report is again that S is undefined rather than that no effect was observed. Both rules are violated in the corpus mapped here, and the second is the specific failure identified in Section 3.7.

A worked application makes the definition concrete, using tier 1 nominal values as reported by the original authors; the application to all six studies of the commensal subset is given in S2.5. For miconazole in the ureasil–polyether ovule [24], CF is the inhibitory concentration for each species, 1.25 µg/mL for *C. tropicalis*, 2.5 µg/mL for *C. parapsilosis*, and 12.5 µg/mL for *C. glabrata*; CE is 5.94 µg/mL, the lowest tested concentration at which VK2/E6E7 viability fell below 40%, a less stringent surrogate than the IC10 specified in Table 2; and CM lies above 95 µg/mL, the concentration at which *L. jensenii* viability fell significantly. The smaller upper bound is CE, so S = 5.94/1.25 = 4.8 for *C. tropicalis* and 5.94/2.5 = 2.4 for *C. parapsilosis*, both open windows constrained by the epithelium (host selectivity ratios 4.8 and 2.4, microbiota selectivity ratios above 76 and 38), whereas for *C. glabrata* S = 5.94/12.5 = 0.48, a closed window reported as such and not as a negative value. The in vitro release of about 135 µg/mL within 24 h lies above CE for every species, so the formulation would be inferred to deliver beyond its window if release concentration were taken as exposure, which is why a tier 2 measurement is required before that inference is drawn. For tea tree oil in the vaginal suppository study [23], CF is 0.25% *v*/*v* and CM is 2% *v*/*v* for the most susceptible lactobacillus, a microbiota selectivity ratio of 8, but CE was not measured, so S is undefined under the first rule: the favourable ratio is reported, and the index is not. These two results, one computable and species-dependent and one undefined, are the outcomes the definition is designed to keep apart from a single summary number.

#### 4.8.2. Which Concentration Is on the Axis

This framework criticizes the use of a MIC without exposure information, and it would reproduce that error if the axis on which its three domains are plotted were left undefined. A concentration applied to a well is not the concentration available to an organism at the mucosa. Between the two lie mucus and protein binding, dilution by cervicovaginal fluid and by the discharge that accompanies the disease being treated, leakage and mechanical loss, the release kinetics of the dosage form, partitioning into tissue and lipid, local metabolism, and the pH dependence of activity for azoles and terpenes alike. The last of these is not one-directional: in a gelatin matrix carrying the same class of phenolic monoterpenes, release and disintegration varied with the pH of the surrounding medium, and the oil itself shifted the pH of that medium, so the vehicle, the local environment, and the compound each act on the other [70]. The tioconazole series in Section 3.3 show the magnitude directly: a roughly twentyfold spread at identical dose within one study, and a difference of more than an order of magnitude in the mean concentration reported at 24 h between two studies of the same drug at the same dose. Two further determinants are physiological rather than pharmaceutical and are not addressed by the corpus mapped here: oestrogen status and menstrual-cycle phase alter glycogen availability, epithelial thickness, fluid volume, and *Lactobacillus* dominance [48], so both the local exposure and the commensal state against which selectivity is judged vary with them, and any tier 2 or tier 3 measurement should record both.

Any use of the framework should therefore declare which of three exposure tiers its values belong to, and a window should be computed only from values of one tier. Tier 1 is the nominal applied concentration, which nearly all in vitro studies report: adequate for ranking candidates, inadequate for asserting that a product operates inside its window. Tier 2 is the measured local concentration in cervicovaginal fluid or tissue, with free and total concentrations distinguished where binding is appreciable. Tier 3 is bioactive exposure at the target over the relevant interval, which for a locally administered agent requires a time course. Only a window whose upper bound is compared against a tier 2 or tier 3 exposure supports the statement that a formulation delivers into it, and no such comparison is currently available for any topical antifungal in routine use, the deficit the instrument in Section 4.10 is intended to address.

#### 4.8.3. Constraints on Interpretation

The host selectivity ratio and the microbiota selectivity ratio defined in Section 4.8.1, and the index derived from them, are ranking instruments only. No universal numeric threshold is currently validated, and none should be inferred from the values reported in individual studies. A candidate with a larger index is a better prospect than one with a smaller index determined under the same predefined experimental specification and exposure tier, and nothing further follows from the number itself.

Three limitations of the concept must be stated explicitly. First, this is not a breakpoint and must not be reported as one; it is a development construct applied to candidates, not an interpretive category applied to isolates. Second, the window is formulation-specific rather than compound-specific, because the exposure profile that determines whether the window is reached is a property of the delivery system. Third, the window is a static representation of a dynamic process: local concentration declines over hours, so a candidate may occupy the window transiently and leave it, which is precisely why formulation-specific exposure data are required rather than nominal concentrations. The relationship between the three domains across the exposure gradient is shown in Figure 4B.

### 4.9. Formulation, Combination Pharmacology, and the Proposed Pathway

For local treatment, formulation is not a passive carrier. Solubility, release kinetics, mucoadhesion, achieved concentration, tissue penetration, and residence time determine biological performance and therefore determine whether the selectivity window described above is reached at all. The same phytochemical may be unsuitable in free form and useful in a controlled delivery system, as the curcumin data illustrate. Translational studies should consequently report formulation-specific exposure rather than the nominal concentration of the active compound, and the finished vehicle should be characterized physicochemically, since the vehicle, the local environment, and the compound act on one another [70].

Formulation research is nevertheless directed away from the products in actual use: within the same 400 records, gels appear in 21 and nanoparticulate or liposomal systems in 20, while ovules, pessaries, and suppositories appear in 11, creams in eight, and vaginal tablets in one. Characterizing the release and retention behaviour of an existing clotrimazole pessary would inform current practice immediately and provide the benchmark against which any novel system should be judged. Combination development should likewise extend beyond checkerboard testing: a fractional inhibitory concentration index identifies a preliminary signal, but promising combinations should progress to time-kill analysis, biofilm testing, epithelial and commensal compatibility, and formulation-specific exposure, advancing only when the antifungal gain clearly exceeds the additional host and ecological cost, which is the selectivity window applied to a two-component system.

The ordering of the pathway follows from this. A preliminary epithelial and ecological screen belongs early, because it identifies candidates that are grossly unselective before resources are committed. The decisive selectivity assessment, however, belongs after combination and formulation work rather than before it, because both reduce the antifungal concentration required and can therefore move a candidate into a window it did not occupy on its own. Applying the gate only to the single compound would discard candidates that the adjunct rationale exists to rescue; applying it only at the end would waste development on compounds that were never plausible.

The framework separates two pathways. The clinical pathway runs from isolate recovery and species identification through standardized AFST, where indicated, to EUCAST or CLSI interpretation where applicable. The translational pathway begins with a topical agent or botanical candidate and proceeds through chemical qualification, quantitative activity, relevant species and resistance phenotypes, biofilm and virulence, epithelial and ecological compatibility, combination benefit, formulation and local exposure, and integrated mucosal models. A MIC alone cannot join them: the proposed point of convergence is reached when susceptibility, local exposure, pharmacodynamic effect, and clinical outcome have been prospectively connected. The staged pathway is shown in Figure 5 and the evidence required at each domain in Table 3.

### 4.10. Measuring Local Exposure: A Proposed Instrument

The most direct way to resolve the topical susceptibility gap is a prospective cohort linking quantitative topical MICs to real treatment exposure and outcome. Such studies are exceptionally scarce: among the 567 susceptibility-related VVC records of Section 3.2, only three mention a cure endpoint together with MIC determination, and one is a perspective article [13]. Feasibility is supported by existing reference-laboratory programmes, in which revised interpretive criteria have reclassified an unchanged collection of 2345 isolates [71], and the need by recent surveillance reporting rising non-albicans yeasts and fluconazole resistance among isolates from women with recurrent VVC [72]. The instrument we propose for the missing quantity is a cervicovaginal antifungal activity titre (CV-AAT): cervicovaginal fluid sampled at defined intervals after administration of a topical agent and titrated against the patient’s own isolate, the mucosal analogue of serum fungicidal concentration testing [12], for which the only precedent is the bioassay of drug in vaginal fluid against a laboratory yeast in the two historical tioconazole series [18,21]. CV-AAT is a proposed assay, not an available one; the minimum clinical dataset, the analytical and clinical validation it would require, the nested cohort that could test whether a topical MIC predicts outcome and whether repeated topical azole exposure shifts MIC distributions, a question that Maillard’s appraisal of topical azole use leaves open for the vaginal setting [73], and the two-tier laboratory model within which the whole programme fits under current EUCAST guidance [39] are set out in Supplementary Note S4. Implementation requires no new susceptibility categories: the innovation lies in generating longitudinal evidence, not in inventing local S/R thresholds.

## 5. Limitations

Several limitations constrain the present conclusions. First, topical antifungal MIC studies use heterogeneous methods, concentration ranges, endpoints, and interpretive labels, which limits comparability across the studies synthesized here. Second, local ECOFFs derived from single populations are not universal thresholds and should not be applied outside the setting in which they were generated. Third, most botanical studies remain dominated by planktonic or biofilm endpoints without equivalent epithelial and microbiota assessment, so the selectivity window can rarely be populated with real numbers at present; it is proposed as a design requirement, not as a summary of existing data. Fourth, essential oils and botanical extracts vary chemically between preparations unless rigorously standardized, and cross-study comparison of an unstandardized oil is of limited value [57]. Fifth, this study is restricted to vulvovaginal candidiasis, and its parameters should not be transferred to oropharyngeal disease without an independent ecological model. Sixth, integrated-domain outcomes occur in single figures, so any change over time in integrated reporting remains undetermined. Seventh, the validation substudy rests on 29 records, so the confidence intervals around the sensitivity estimates are wide, and full text could not be obtained for 11 of the 40 sampled records; if inaccessible articles differ systematically from accessible ones, those estimates are biased to an unknown degree; the substudy was also drawn from the original 175-record subset, so the records added after the term-filter check were not sampled. Eighth, the corpus was assembled by an automated term filter rather than by human screening, which makes it reproducible but only as sensitive as its term set. The check reported in Section Sensitivity of the Plant-Derived Term Filter found that the original 14 terms captured 54.5% of the final corpus; the extension recovered the remainder that an expanded sweep and dual human screening could identify, and the residual false-negative rate among records matched by no term is estimated at 1.3% (95% CI 0.2 to 4.7%), so a number of plant-derived records in the low tens, and possibly more, remain unidentified. The database searches, the full-text recoding of the validation substudy, and the eligibility judgements for the narrative synthesis remained single-reviewer tasks. The prevalence estimates should therefore be read as properties of the corpus as assembled rather than as unbiased estimates of the underlying literature, and the comparisons between domains, which the extension did not alter, are the more robust quantity. Ninth, domain-level inference was confined to the Web of Science corpus, a design decision rather than a prespecified hypothesis, and since no single database contained more than approximately two-thirds of the pooled set the analytic corpus is a subset of the retrievable literature. The prespecified sensitivity analysis (S1.10, Section Cross-Corpus Sensitivity Analysis) found the ordering of domains preserved in an independently retrieved Scopus corpus, which reduces but does not remove the concern: it rests on term matching rather than consensus coding and could not be extended to PubMed. The ordering is therefore attributable to two corpora, not yet to the field, and replication with full consensus coding on a second corpus remains the most informative outstanding check. Tenth, because abstract-level coding missed evidence in four of the five domains, the absolute prevalences are lower bounds for those domains; the comparisons between domains are the interpretable quantity, and even those assume that the sensitivity differences observed in the validation sample are not large enough to reverse the ordering. Eleventh, the framework proposed in Section 4.8 is a theoretical construction motivated by the mapping and by the exposure literature, not a result derived from either; it has not been applied prospectively to any candidate, and the conventions specified in Table 2 have not been validated or agreed by consensus. Its status is hypothesis-generating, and the rules given there are intended to be tested and revised rather than adopted. Twelfth, the mapping does not stratify by geography or by host population: it cannot say whether the pattern differs between regions, whether climate-related shifts in species distribution affect it, or whether the candidates discussed behave differently in women living with HIV, transplant recipients, or patients receiving cancer therapy, in whom vulvovaginal candidiasis is more often complicated and in whom every parameter of the framework would require separate definition. Nor does it address oestrogen status or menstrual-cycle phase, which affect both local exposure and the commensal state (Section 4.8.2). Finally, this is a critical translational synthesis rather than a formal systematic review or meta-analysis; its purpose is to define a development framework, not to estimate pooled treatment effects.

## 6. Conclusions

The increasing rigor of AFST should not be interpreted as creating a diagnostic void in vulvovaginal candidiasis. It clarifies the limits of an older approach in which growth inhibition by a topical agent was translated too readily into a clinically predictive susceptible or resistant result. Reference EUCAST and CLSI methodologies should remain the basis of clinical AFST, and categorical interpretation should be restricted to organism and drug combinations for which validated criteria exist.

Quantitative testing of topical antifungals remains scientifically valuable, but its immediate role is epidemiological surveillance, detection of unusual phenotypes, and prospective correlation with local treatment outcome. The absence of a topical breakpoint should not be filled by a surrogate breakpoint. It should be filled by better data.

Plant-derived compounds belong in this translational space only when developed as standardized adjuncts. Their value should be judged by selective antifungal or anti-virulence gain relative to epithelial and microbiota cost, not by MIC alone. Tea tree oil and terpinen-4-ol provide a benchmark for integrated assessment, thymol illustrates combination-oriented development, curcumin illustrates formulation-dependent co-delivery, and geraniol illustrates anti-virulence development. None can yet be described as a validated microbiota-sparing treatment for VVC.

The central research question is therefore no longer whether a botanical extract is antifungal. Standard AFST tells us what the organism does under reference conditions; topical translational testing must determine whether effective mucosal exposure exists within a window that suppresses *Candida* while preserving the epithelium and the protective microbiota. Defining, measuring, and reporting that window is the work the field now needs.

Stated precisely, and within the limits set out in Section 5, this study demonstrates an evidence deficit in a defined and reproducibly retrieved corpus and proposes, in response to it, a framework that now requires prospective validation. The mapping is the evidence; the window is the hypothesis, offered with an operational definition so that it can be tested and, if necessary, refuted. The practical finding for a clinician or a developer reading no further is simpler than either: for locally administered antifungal candidates in vulvovaginal candidiasis, testing against the protective commensals is essentially absent from the literature, and a product should not be called microbiota-sparing until that test has been done on the same concentration scale as its antifungal effect.

Qualifying detail: tea tree oil is a standardized oil with documented batch variability, and its *Lactobacillus* evidence rests on a limited panel in a single formulation study. Terpinen-4-ol data cover azole-susceptible and azole-resistant isolates. Curcumin evidence derives from two successive miconazole–curcumin ovule systems, with *L. jensenii* tested within the intended formulation. Geraniol appears in only four VVC-indexed records and its in vivo data are murine and combined with vaginal washing. Eugenol has evidence volume comparable to thymol; its commensal margin was measured against *L. acidophilus*, which is not a vaginally dominant species, and no vaginal epithelial model has been used. Carvacrol and eugenol show concentration-dependent host toxicity.

All three concentrations should be determined over a harmonized concentration series and matched exposure duration, using model-appropriate media under vaginally relevant conditions, and at the same declared exposure tier; where matrix or pH cannot be identical, bridging controls should be used and cross-assay ratios reported as exploratory. The specification is proposed for consensus and testing; it is not a validated standard, and none of the quantities defined here is a clinical breakpoint.

## Figures and Tables

**Figure 1:**
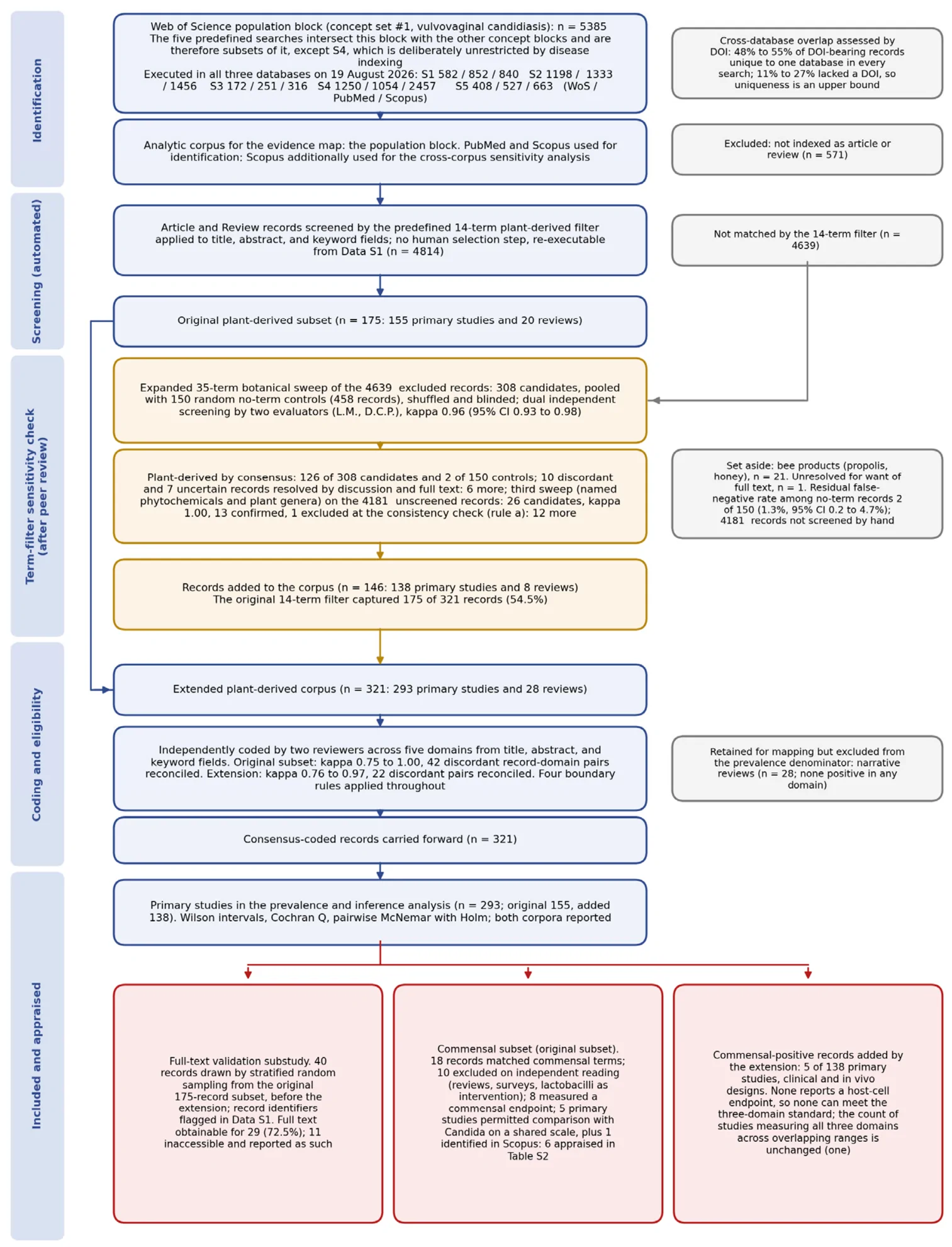
Flow of sources of evidence through the study, in the PRISMA-ScR band structure, with the reason and count given for every exclusion. Retrieval counts are shown for each of the five searches in each database; the population block is the vulvovaginal candidiasis concept set, of which S1, S2, S3, and S5 are subsets and S4, unrestricted by disease indexing, is not, so the block is larger than any single search and is not their sum. Cross-database overlap was assessed by DOI; records without a DOI could not be matched across sources, so uniqueness estimates are upper bounds. The Web of Science population block is the analytic corpus and served as the denominator for the evidence mapping, while PubMed and Scopus were used for identification and for the overlap analysis, and Scopus was additionally analysed in the cross-corpus sensitivity analysis; neither was pooled into the primary domain-level inference. All 321 plant-derived records, the original 175 and the 146 added after the term-filter check, were consensus-coded, while prevalence estimates and inference use the 293 primary studies within that set and are reported alongside the original 155. The plant-derived subset was extracted automatically by the predefined term set, with no human selection step; its sensitivity was tested after peer review by dual independent screening and the subset was extended (Section Sensitivity of the Plant-Derived Term Filter), and duplicate independent coding was applied to every record of the plant-derived subset. The two branches at the foot of the diagram are the full-text validation substudy and the commensal subset appraised in detail. The completed PRISMA-ScR checklist is provided in S1.9.

**Figure 2:**
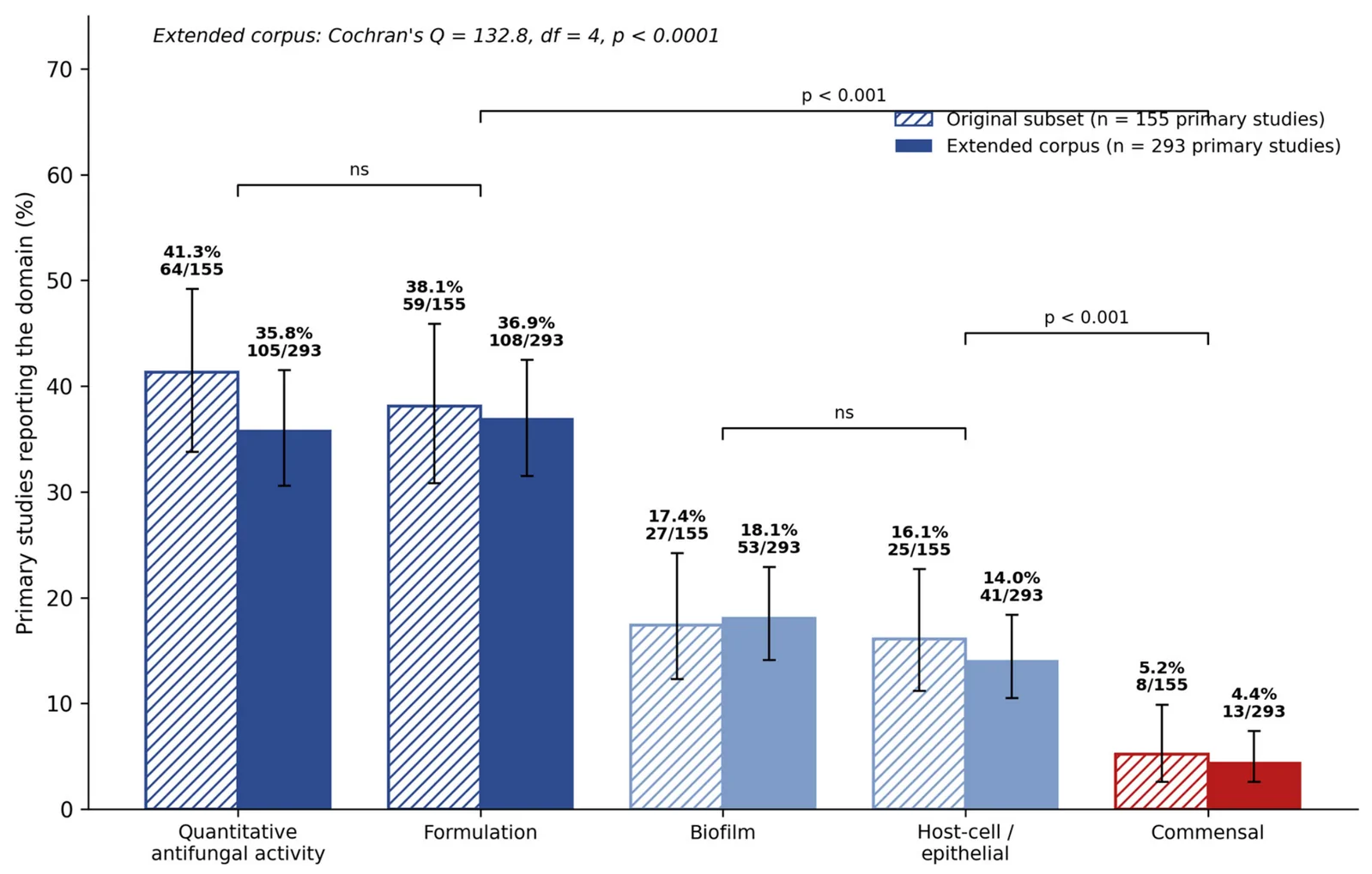
Domain coverage among primary studies in the plant-derived subset (*n* = 155; consensus coding of two independent reviewers). Bars show the percentage of primary studies reporting each domain, with 95% Wilson confidence intervals. Brackets show selected Holm-adjusted pairwise McNemar comparisons; the full set of ten comparisons is reported in Section 3.4 and in Data S1. ns denotes *p* = 1.0. Quantitative activity and formulation do not differ from each other, biofilm and host-cell coverage do not differ from each other, and commensal testing is significantly lower than every other domain. The 20 reviews in the subset contributed no positive code in any domain and are excluded from the denominator; proportions across all 175 records are given in the text.

**Figure 3:**
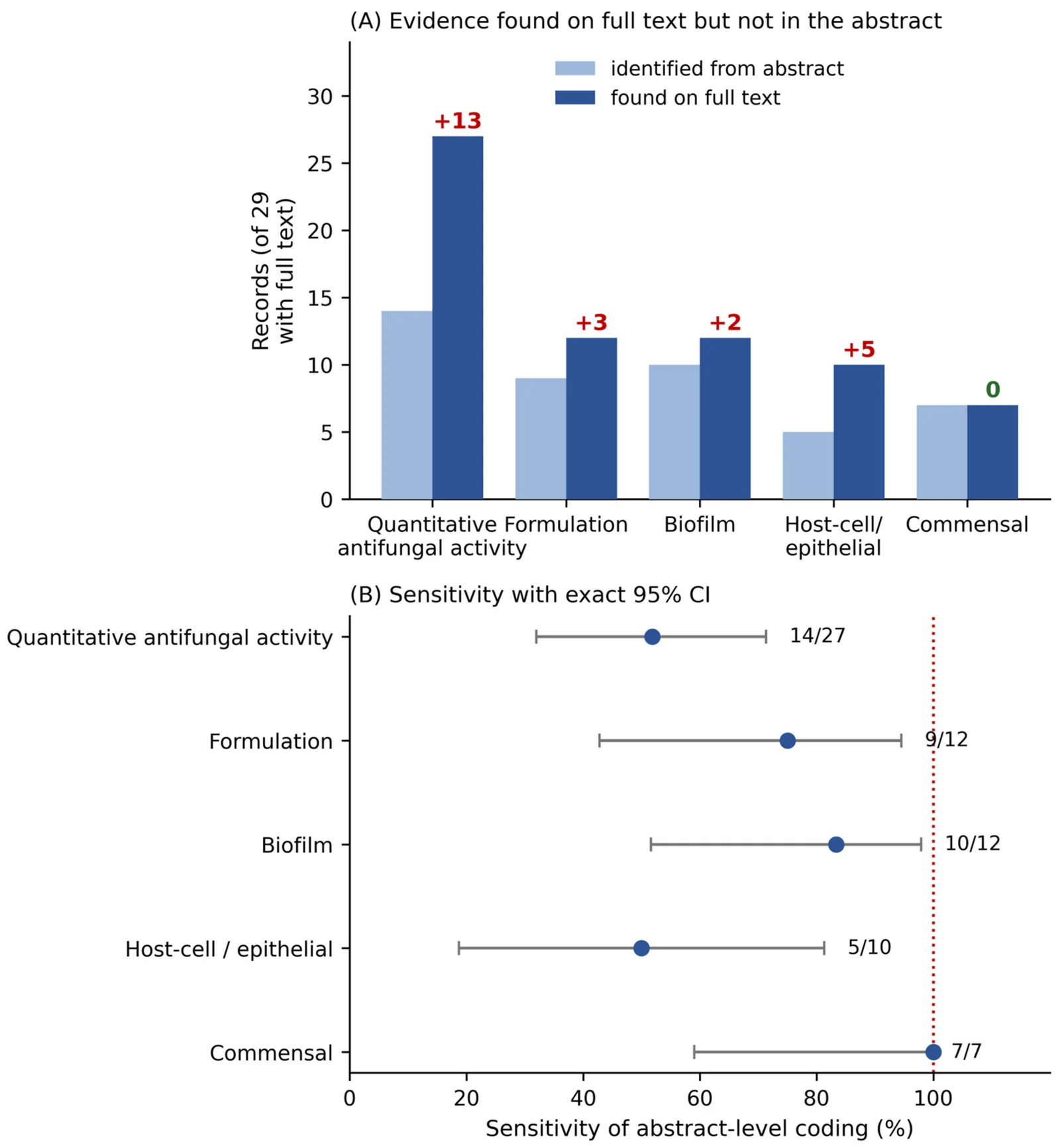
Full-text validation substudy (29 records with obtainable full text, of 40 sampled). (**A**) Records identified from the abstract compared with records found on full-text reading; red values are false negatives. Abstract-level coding produced 23 false negatives and no false positives across the 145 record-domain pairs; discordance is reported descriptively because multiple domains were evaluated within the same records. (**B**) Sensitivity of abstract-level coding by domain, with exact binomial 95% confidence intervals; the dotted vertical line marks 100% sensitivity. No missed commensal evidence was observed in this validation sample, although the small number of positive records limits precision.

**Figure 4:**
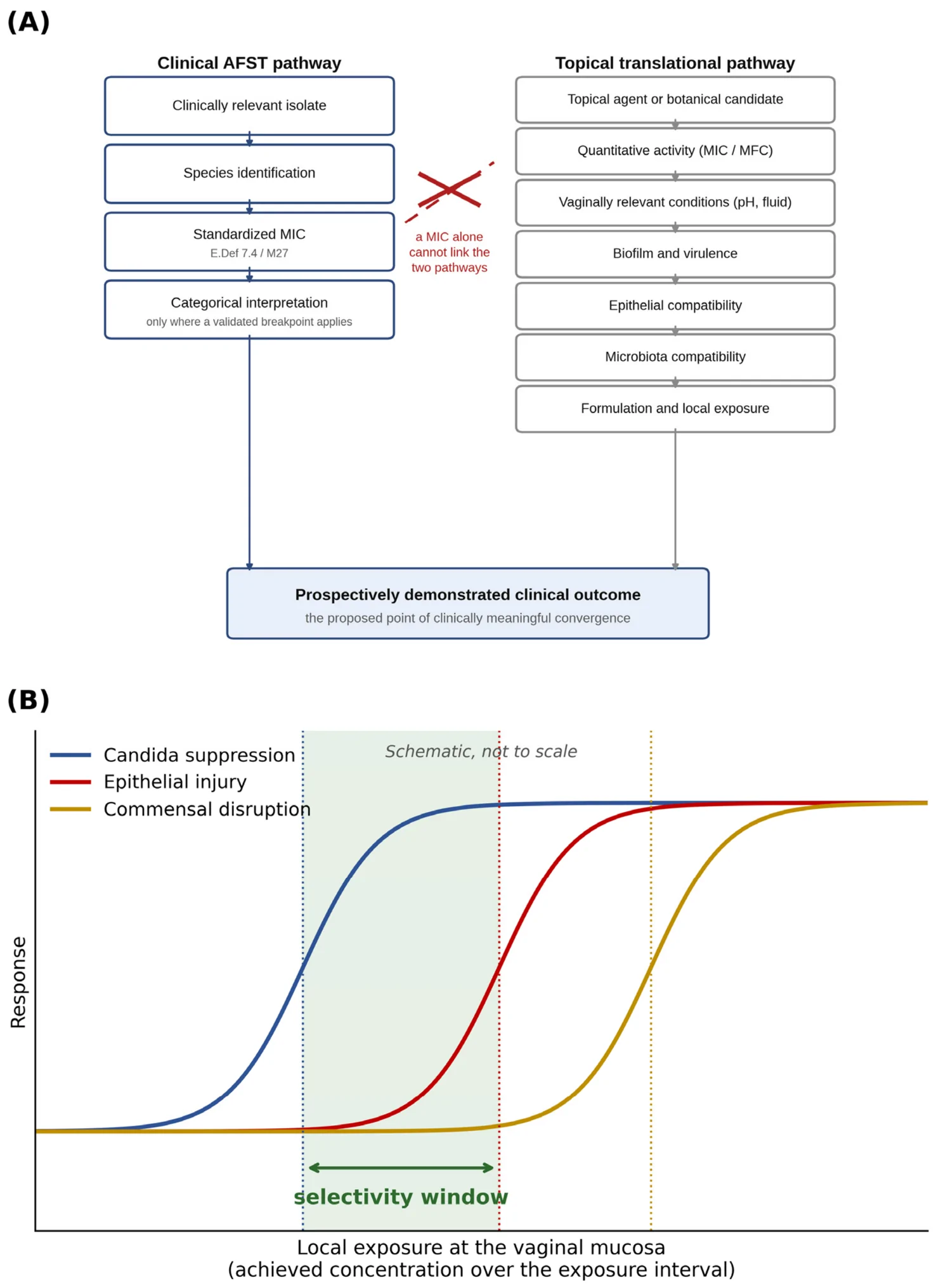
The topical susceptibility gap and the mucosal antifungal selectivity window. (**A**) Standardized clinical AFST proceeds from species identification to quantitative MIC determination, and to categorical interpretation only where a current validated breakpoint applies; topical translational testing proceeds from quantitative activity to vaginally relevant conditions, biofilm, epithelial compatibility, microbiota compatibility, formulation, local exposure, and prospective outcome. Both pathways generate MIC values, but a MIC alone cannot link them, because it fixes neither the exposure achieved at the mucosa nor the cost to the epithelium and the resident microbiota; the proposed point of convergence is prospectively demonstrated outcome. (**B**) The horizontal axis represents local exposure at the vaginal mucosa, expressed as achieved concentration over the exposure interval rather than as nominal formulation strength; three response bands are plotted on this common axis, suppression of *Candida* growth or virulence, epithelial injury, and disruption of protective commensals, and the selectivity window is the interval in which fungal suppression is achieved before the epithelial and commensal effect concentrations are reached. A wide window indicates tolerance of formulation and clearance variability; a narrow or absent window indicates overlapping exposures. Both panels are schematic and state a development proposal: the curves do not represent measured data, and the concept must not be interpreted as a clinical breakpoint or as a validated numeric safety index.

**Figure 5:**
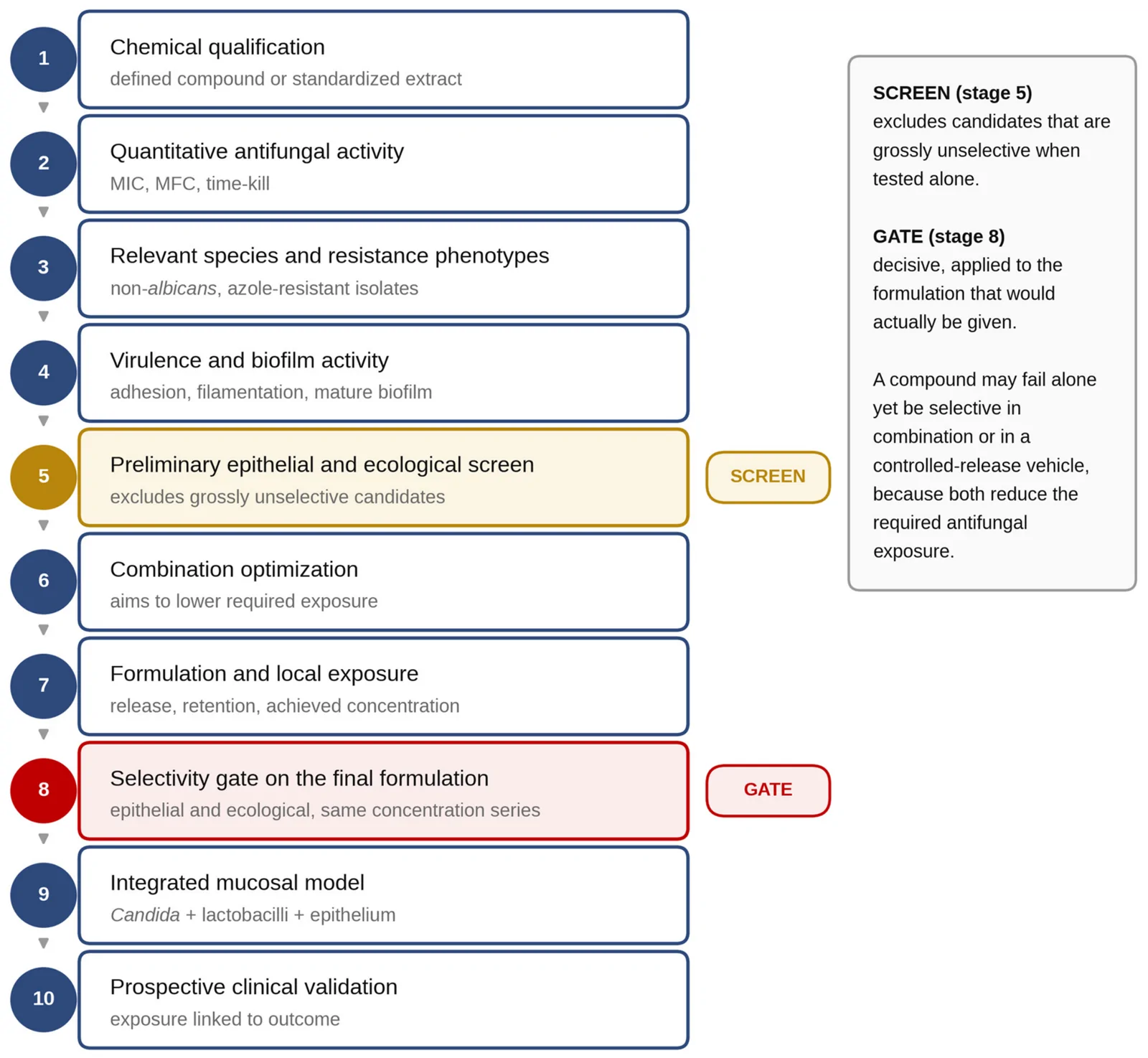
Developmental cascade for locally administered antifungal adjuncts. Chemical qualification, then quantitative antifungal activity, then relevant species and resistance phenotypes, then virulence and biofilm activity, then a preliminary epithelial and ecological safety screen, then combination optimization with a conventional antifungal, then formulation and local exposure, then a definitive epithelial and ecological selectivity gate applied to the final formulation, then an integrated *Candida*, lactobacillus, and epithelium model, and finally prospective clinical validation. The early screen and the late gate are deliberately separated. A compound that appears unfavourable in isolation may become selective in combination or in a controlled-release vehicle, since both reduce the antifungal concentration required, so a candidate should not be discarded on single-agent data alone; conversely, no candidate should proceed to clinical evaluation without selectivity demonstrated for the formulation that would actually be administered.

**Table 1:** Evidence matrix for selected plant-derived candidates in vulvovaginal candidiasis.

Candidate	Chem.	Anti-*Candida*	NAC	Biofilm	Epith.	*Lacto.*	Form.	Position
*Melaleuca alternifolia* oil	●●	●●●	●●	●●	●●	●	●●	Translational benchmark
Terpinen-4-ol	●●●	●●●	●●●	●●	●	●	●●	Defined constituent benchmark
Thymol	●●●	●●●	●	●●●	●●		●●	Combination candidate
Curcumin	●●	●●	●●	●●	●●	●●	●●●	Formulation-driven; only entry with commensal and antifungal data across overlapping concentration ranges in one formulation system
Geraniol	●●●	●	●	●●	●		●●	Anti-virulence; least documented
Carvacrol	●●●	●●●	●	●●●	●		●	Secondary candidate
Eugenol	●●●	●●	●●	●●	●	●●	●●	Secondary; commensal margin documented, host toxicity reported, vaginal epithelial model absent

The ordinal marks are not a validated scoring instrument; a difference of one mark should not be read as a quantitative difference in evidence. Scoring: ●●● multiple independent studies, including clinically relevant isolates or a formulation-specific model; ●● at least one primary study under conditions relevant to vaginal exposure; ● preliminary or single-study evidence; no mark, domain not investigated under relevant conditions (not a negative result and not evidence of safety). The study-level appraisal underlying each mark, with the qualifying detail summarized here, is given in Table S2.

**Table 2:** Proposed operational definition of the mucosal antifungal selectivity window.

Component	Primary Metric	Acceptable Alternatives	Reporting Requirements
Fungal effective concentration (CF)	MIC by EUCAST E.Def 7.4 or CLSI M27, endpoint and reading time stated	MFC; MBIC or MBEC where the target is biofilm; a defined log10 reduction; EC50 for a named virulence trait such as filamentation	Species and strain identity; inoculum; medium and pH; incubation time; whether the endpoint is inhibitory, fungicidal, or anti-virulence. Inhibitory and anti-virulence endpoints must not be combined in one index unless both are reported.
Epithelial threshold concentration (CE)	Concentration producing 10% loss of viability (IC10) in a reconstructed human vaginal epithelium, exposure matched to the intended dosing interval	LDH release above a stated multiple of control; loss of transepithelial electrical resistance above a stated threshold; IC50 in a vaginal epithelial monolayer, declared as a less stringent surrogate	Model type (monolayer or reconstructed tissue); exposure duration; readout and threshold used; the full concentration range tested, and explicitly whether it spans CF. IC10, IC50, LDH release, and TEER loss are not interchangeable and are not crossed at the same concentration.
Commensal threshold concentration (CM)	Lowest concentration producing a 1 log10 reduction in viable count for the most susceptible species in a declared vaginal *Lactobacillus* panel	MIC for the declared panel; loss of a stated metabolic function such as lactate or hydrogen peroxide production; shift in relative abundance in a defined community model	Species, strain provenance, and panel composition; *L. crispatus* required as a minimum, with *L. gasseri* or *L. jensenii* preferred in addition; growth conditions, medium, and pH, which must be vaginally relevant; whether the commensal was exposed alone or in co-culture. Non-vaginal species such as *L. casei* may be reported but do not satisfy the requirement.
Exposure axis and tier	Measured local concentration in cervicovaginal fluid or tissue across the dosing interval (tier 2)	Concentration released from the finished dosage form under stated sink conditions; nominal applied concentration, declared as tier 1	Tier declared explicitly; free versus total concentration; matrix and pH; sampling times. A window may be computed only from values of a single tier, and only a tier 2 or tier 3 comparison supports the claim that a formulation delivers into the window.
Derived quantities	Selectivity index S, the ratio of the smaller of CE and CM to CF	Host selectivity ratio (CE/CF) and microbiota selectivity ratio (CM/CF), reported alongside S to identify the constraining domain	S greater than 1 denotes an open window; S of 1 or less denotes a closed window and is reported as such, not as a negative value; S is undefined where a protected domain was untested or was tested only below CF. No validated threshold value of S exists.

**Table 3:** Minimum translational framework for vaginal adjunct development.

Domain	Minimum Evidence	Preferred Advanced Evidence
Chemical identity	Defined compound or characterized standardized extract	Batch-to-batch analytical fingerprint
*Candida* panel	*C. albicans* + relevant non-*albicans* species	Large clinical collection stratified by resistance phenotype
Conventional comparators	Topical azole + fluconazole phenotype	Add nystatin and multiple local agents
Growth activity	MIC	MIC + MFC + time-kill
Virulence	Adhesion or filamentation	Multiple functional endpoints
Biofilm	Early or mature biofilm assay	Biomass + metabolic activity + viable counts
Host safety	Relevant epithelial viability	Barrier integrity + inflammatory endpoints + repeated exposure
Microbiota	*L. crispatus*, *L. gasseri*, *L. jensenii* under antifungal test conditions	Multispecies community model; separate *L. iners* analysis
Combination	Checkerboard	Time-kill + biofilm confirmation
Formulation	Defined vehicle and concentration	Release kinetics + mucoadhesion + local exposure
Integrated model	Not required at entry	*Candida* + lactobacilli + epithelium
Translation	Relevant in vivo model when justified	Prospective human tolerability, ecology, efficacy, and recurrence

## Data Availability

The record-level coding dataset, the analysis scripts, and their outputs are provided as Supplementary Data S1. The principal inferential statistics for the evidence-mapping and full-text validation analyses, together with the cross-corpus sensitivity analysis, are reproduced by the supplied scripts and datasets. The raw database exports required to run the cross-corpus sensitivity routine, comprising the Web of Science population block, the Scopus plant-derived export, and the PubMed plant-derived export, are provided with it. No other data were generated or analysed.

## Supplementary Materials

The following supporting information can be downloaded at: https://www.mdpi.com/article/10.3390/jof12100722/s1, Table S1: Search strategy, evidence selection, and coding (concept blocks as executed, record counts across the three databases, coding dictionary, derivation of every composite count, duplicate coding and adjudication rules, PRISMA-ScR checklist (S1.9), the prespecified protocol and results of the cross-database sensitivity analysis (S1.10), and the term-filter sensitivity check and extension of the plant-derived subset (S1.11)); Table S2: Appraisal of primary plant-derived studies testing both *Candida* and vaginal commensals; Supplementary Note S3: Laboratory interpretive practice for vaginal *Candida* isolates, with Table S3: Proposed laboratory terminology for vaginal *Candida* isolates, Table S4: Antifungal agents relevant to vulvovaginal candidiasis: analytical value and interpretive limits, and Figure S1: Computing the selectivity index; Supplementary Note S4: Measuring local exposure: the proposed instrument in full; Data S1: Record-level dataset, analysis script, and output (coding_dataset.csv and coding_dataset_extended.csv; analysis_S1.py, analysis_S1_extended.py, sensitivity_S1.py and screening_sweep_S1.py with their outputs; the dual-screening and coding workbooks of Section Sensitivity of the Term Filter and Extension of the Corpus; and the raw Web of Science, Scopus, and PubMed exports required to run them).
