# Supplementary material for: Stepped Evidence Coverage for Plant-Derived Adjuncts in Vulvovaginal Candidiasis: An Evidence Map and a Proposed Mucosal Antifungal Selectivity Window
Source: J Fungi (Basel). 2026 Sep 24;12(10):722. doi: 10.3390/jof12100722 (PMC13706402; doi:10.3390/jof12100722)
Supplement: Supplementary file 1 [file jof-12-00722-s001.zip › Supplementary_Material.pdf]

Supplementary Material

Stepped Evidence Coverage for Plant-Derived Adjuncts in Vulvovaginal Candidiasis: An Evidence Map and a Proposed Mucosal Antifungal Selectivity Window

Contents

Item	Content
Table S1	Search strategy, concept blocks as executed, record counts across three databases, coding dictionary, derivation of every composite count, duplicate coding and adjudication rules, PRISMA-ScR checklist with stated departures, and the prespecified protocol for the cross-database sensitivity analysis
Table S2	Study-level appraisal of the primary plant-derived studies testing both *Candida* and vaginal commensals
Data S1	Record-level coding dataset, analysis script, and machine-readable output
Note S3	Laboratory interpretive practice moved from the main text after peer review: Table S3 (proposed laboratory terminology), Table S4 (antifungal agents relevant to vulvovaginal candidiasis), and Figure S1 (computation of the selectivity index)
Note S4	Measuring local exposure: the proposed instrument in full (moved from Section 4.10 of the first submitted version)

The principal inferential statistics for the evidence-mapping and full-text validation analyses, together with the cross-corpus sensitivity analysis, are reproduced by the scripts and datasets in Data S1: the primary analysis from the record-level coding dataset, and the cross-corpus sensitivity analysis of Section 3.4.1 from the raw database exports, both provided. Counts obtained by automated term matching, which are used only to describe the composition of the wider literature and are explicitly reported as upper bounds, are produced by a separate routine documented in S1.7.

Table S1. Search strategy, evidence selection, and coding

S1.1. Databases, dates, and execution

Item	Specification
Databases	PubMed (MEDLINE), Web of Science Core Collection, Scopus

Item	Specification
Coverage	Database inception to 19 August 2026
Date of last search	19 August 2026 (all three databases executed on the same day)
Editions searched (Web of Science Core Collection)	All editions available to the subscribing institution: SCI-EXPANDED, SSCI, A&HCI, ESCI, CPCI-S, CPCI-SSH, BKCI-S, BKCI-SSH, CCR-EXPANDED, IC. Coverage years as provided by the institutional subscription; timespan set to all years
Language	No restriction at retrieval or screening; records in languages other than English were retained (62 such records in the Scopus population set alone). Full-text assessment in English, with translation where required to determine eligibility
Publication types	Primary experimental studies, clinical studies, guidelines, methodological standards; narrative reviews used for context only
Additional sources	EUCAST, CLSI, CDC, ACOG, ECMM/ISHAM/ASM organizational documents retrieved directly from the issuing bodies; manual screening of reference lists of included articles
Deduplication	Programmatic, on DOI, across the three database exports. Between 11% and 27% of records per database carried no DOI; these could not be matched across sources and were treated as distinct, so the proportion reported as unique to a single database is an upper bound
Screening	Records were screened by title and abstract, and assessed at full text, by a single reviewer. No dual independent screening was performed; this is stated as a limitation in the main text

All searches should be executed on the same day and exported as .ris or .nbib with the record count captured at the moment of export, because counts drift over weeks.

S1.2. Concept blocks

The strategy uses five concept blocks combined into five searches. Blocks are written for PubMed; equivalents for Web of Science and Scopus follow in the Results and Discussion.

Block A. Population (vulvovaginal candidiasis)

```
"Candidiasis, Vulvovaginal"[Mesh] OR "vulvovaginal candid*"[tiab] OR
"vaginal candid*"[tiab] OR "vulvovaginitis"[tiab] OR "vaginal thrush"[tiab]
OR
```

```
(Candida[tiab] AND (vagina*[tiab] OR vulvovagina*[tiab] OR
cervicovagina*[tiab]))
```

Block B. Susceptibility testing and interpretation

```
"Microbial Sensitivity Tests"[Mesh] OR "antifungal susceptibility"[tiab] OR
AFST[tiab] OR "minimum inhibitory concentration*"[tiab] OR MIC[tiab] OR
MFC[tiab] OR
"epidemiological cut-off"[tiab] OR "epidemiological cutoff"[tiab] OR
ECOFF[tiab] OR
"clinical breakpoint*"[tiab] OR breakpoint*[tiab] OR EUCAST[tiab] OR
CLSI[tiab] OR
"broth microdilution"[tiab] OR "time-kill"[tiab] OR checkerboard[tiab] OR
"fractional inhibitory concentration"[tiab]
```

Block C. Biofilm, microbiota, and epithelium

```
"Biofilms"[Mesh] OR biofilm*[tiab] OR "Lactobacillus"[Mesh] OR
Lactobacillus[tiab] OR
crispatus[tiab] OR gasseri[tiab] OR jensenii[tiab] OR iners[tiab] OR
"vaginal microbiota"[tiab] OR "vaginal microbiome"[tiab] OR "vaginal
flora"[tiab] OR
"community state type*"[tiab] OR "colonization resistance"[tiab] OR
"vaginal epitheli*"[tiab] OR "epithelial cell*"[tiab] OR cytotoxic*[tiab] OR
"barrier integrity"[tiab]
```

Block D. Plant-derived compounds

```
"Plant Extracts"[Mesh] OR "Oils, Volatile"[Mesh] OR "Phytochemicals"[Mesh] OR
"essential oil*"[tiab] OR "plant extract*"[tiab] OR phytochemical*[tiab] OR
"plant-derived"[tiab] OR botanical*[tiab] OR "tea tree"[tiab] OR
Melaleuca[tiab] OR
"terpinen-4-ol"[tiab] OR thymol[tiab] OR carvacrol[tiab] OR curcumin[tiab] OR
geraniol[tiab] OR eugenol[tiab] OR terpene*[tiab]
```

Block E. Locally administered antifungal agents and formulation

```
clotrimazole[tiab] OR miconazole[tiab] OR econazole[tiab] OR
fenticonazole[tiab] OR
sertaconazole[tiab] OR butoconazole[tiab] OR tioconazole[tiab] OR
nystatin[tiab] OR
fluconazole[tiab] OR "topical antifungal*"[tiab] OR
"Administration, Intravaginal"[Mesh] OR intravaginal[tiab] OR
pessary[tiab] OR ovule*[tiab] OR suppositor*[tiab] OR "vaginal cream*"[tiab]
OR
"vaginal gel*"[tiab] OR "vaginal film*"[tiab] OR mucoadhesi*[tiab] OR
"drug delivery"[tiab] OR "release kinetic*"[tiab]
```

S1.3. The five searches

Concept blocks as executed in Web of Science, 19 August 2026

Set	Block	Records
#1	Population (vulvovaginal candidiasis)	5,385
#2	Susceptibility testing and interpretation	201,818
#3	Biofilm, microbiota, epithelium (initial)	1,117,829
#5	Biofilm, microbiota, epithelium (narrowed; generic epithelial and cytotoxicity terms removed)	423,042
#6	Plant-derived compounds	362,664
#7	Local agents and formulation (initial)	348,742
#8	Local agents and formulation (narrowed; generic drug-delivery terms removed)	89,107
#9	Organism (*Candida* and related genera)	132,922

Two blocks were narrowed after inspection of their unrestricted result counts. In each case the removed terms were generic to biomedicine rather than specific to the vaginal niche: “epithelial cell*” and cytotox* in the ecological block, and”drug delivery” and nanoparticle in the formulation block. Both the original and the narrowed versions are reported here; the narrowed versions were used in the combinations below.

Combined searches, executed in all three databases on 19 August 2026

#	Combination	Purpose in the review	Web of Science	PubMed	Scopus	Unique DOIs across the three	Records in only one database
S1	Population AND susceptibility	Topical susceptibility gap	582 (567 articles/reviews)	852	840	1,148	622 (54%)
S2	Population AND ecology	Biofilm, microbiota, epithelium	1,198 (1,125)	1,333	1,456	1,916	1,027 (54%)
S3	Population AND botanicals	Plant-derived compounds in VVC	172 (167)	251	316	363	175 (48%)

#	Combination	Purpose in the review	Web of Science	PubMed	Scopus	Unique DOIs across the three	Records in only one database
S4	Botanicals AND organism AND ecology	Selectivity evidence, deliberately unrestricted by disease indexing	1,250 (1,208)	1,054	2,457	2,679	1,486 (55%)
S5	Population AND local agents AND susceptibility	Agent-specific and formulation-specific exposure	408 (400)	527	663	791	397 (50%)

Deduplication was performed on DOI. Across the five searches, between 48 and 55 per cent of retrieved records appeared in only one of the three databases, and no database contained more than approximately two-thirds of the pooled set for any search. Running all three was therefore necessary for identification rather than confirmatory.

Two invalidated runs, retained for transparency

The first execution of the population block in PubMed returned 977 records because a Review article-type filter persisted from an earlier session; the block was re-run without filters and returned 8,179. In Scopus, an initial attempt to combine sets using Web of Science set notation returned 69,431,785 records, because Scopus does not resolve that syntax; the combination was re-executed using full query strings.

Term-based evidence mapping

Block-level counts and the domain-coverage figures reported in the Results and Discussion derive from the Web of Science export alone (population block: 5,385 records, 4,814 articles and reviews). A single database was used for these counts because field structure, export content, and the fields interrogated differ between the three sources, so mention counts are not comparable across them. PubMed and Scopus were used for identification of literature and were not used for primary mapping enumeration; Scopus was subsequently analysed separately in the prespecified cross-corpus sensitivity analysis.

S1.4. Equivalents for Web of Science and Scopus

Web of Science Core Collection, Topic field:

```
TS=("vulvovaginal candid*" OR "vaginal candid*" OR (Candida NEAR/3 vagina*))
AND TS=("antifungal susceptibility" OR "minimum inhibitory concentration" OR
MIC OR
```

```
ECOFF OR "epidemiological cut-off" OR breakpoint* OR EUCAST OR CLSI OR
"broth microdilution")
```

Scopus, TITLE-ABS-KEY field:

```
TITLE-ABS-KEY("vulvovaginal candid*" OR "vaginal candid*" OR (Candida W/3
vagina*))
AND TITLE-ABS-KEY("antifungal susceptibility" OR "minimum inhibitory
concentration" OR
MIC OR ECOFF OR breakpoint* OR EUCAST OR CLSI OR "broth microdilution")
```

The remaining four searches are translated by the same rule: replace each MeSH term with its free-text equivalents, keep the truncation, and replace PubMed proximity with NEAR/n in Web of Science and W/n in Scopus. Record the exact string actually executed in each database, not the PubMed original.

S1.5. Eligibility criteria

These criteria governed the narrative synthesis and the candidate-level quantitative appraisal. They did not define the mapped corpus: the analytic subset of 175 records comprises Article and Review records within the Web of Science population block that matched the predefined plant-derived term set, extended to 321 records after the term-filter sensitivity check described in Section 2.3.1 of the main text and in S1.11, and retained irrespective of the number of experimental domains subsequently coded, as set out in Section 2.3 of the main text and shown in Figure 1.

Inclusion

- Primary experimental studies reporting quantitative antifungal activity against *Candida* spp. with a defined concentration scale
- Clinical studies of vulvovaginal candidiasis reporting microbiological or clinical outcome
- Studies reporting biofilm, virulence, epithelial, or commensal endpoints relevant to vaginal exposure
- Formulation studies reporting release, mucoadhesion, tissue distribution, or local exposure
- Methodological standards and clinical guidelines from EUCAST, CLSI, CDC, ACOG, IDSA, and ECMM/ISHAM/ASM

Exclusion

- Test material chemically undefined and not reproducibly characterized
- Antifungal activity reported without a concentration scale, for example zone diameters only, with no MIC or MFC
- Models with no relevance to mucosal exposure, for example agricultural or food preservation applications, unless the mechanistic finding is used only for context
- Systemic candidiasis without mucosal relevance
- Conference abstracts without a retrievable full text, and non-peer-reviewed preprints
- Narrative reviews as the sole source of a numerical claim

S1.6. Evidence appraisal domains

Each botanical candidate was appraised across seven domains, scored as reported in Table 1 of the main text and defined in Table S2: chemical definition and reproducibility; quantitative antifungal activity; activity against non-*albicans* or azole-resistant isolates; biofilm or virulence effects; epithelial compatibility; ecological compatibility with vaginal commensals; and formulation or in vivo vaginal progression. Absence of data in a domain was recorded as an evidence gap and never as a negative result or as evidence of safety.

S1.7. Coding dictionary for the term-based evidence mapping

Counts reported in the main text were generated by regular-expression matching against the concatenated title, abstract, author keywords, and Keywords Plus fields of the Web of Science export. A record was counted when any listed term matched. Matching is case-insensitive; * denotes truncation in the description below and was implemented as a regular-expression wildcard.

Coded domain	Terms matched	Reported in
Quantitative antifungal activity	antifungal susceptibilit*, susceptibility test*, minimum inhibitory, MIC (word-bounded), MICs, MFC, epidemiological cut*, ECOFF, clinical breakpoint*, breakpoint*, EUCAST, CLSI, broth microdilution, time-kill, checkerboard, fractional inhibitory	Results, Discussion
Commensal / microbiota	lactobacill*, crispatus, gasseri, jensenii, iners, vaginal microbiot*, vaginal microbiom*, vaginal flora, community state type*, colonis/zation resistance	Results, Discussion
Host-cell / epithelial endpoint	epitheli*, keratinocyte, cell viabilit*, cytotox*, fibroblast, barrier integrit*, HeLa, VK2	Results, Discussion
Biofilm	biofilm*	Results, Discussion
Polymicrobial or interkingdom design	polymicrobial, mixed-species, mixed biofilm, interkingdom, inter-kingdom, dual-species, co-culture, coculture	Results, Discussion
Plant-derived compound	essential oil*, plant extract*, phytochem*, plant-derived, botanical*, tea tree, Melaleuca, terpinen-4-ol, thymol, carvacrol, curcumin, geraniol, eugenol, terpene*	Results, Discussion

Coded domain	Terms matched	Reported in
Explicit concentration scale	MIC (word-bounded), minimum inhibitory, inhibitory concentration, IC50, MFC, MBC, dose-dependent, concentration-dependent	Results, Discussion
Formulation	ovule*, pessar*, suppositor*, *vaginal cream*, vaginal gel*, *vaginal film*, mucoadhes*, drug delivery, nanoparticle, release kinetic*, liposom*	Results, Discussion
Local exposure measurement	cervicovaginal fluid, vaginal fluid, tissue concentration, tissue distribution, pharmacokinetic*, residence time, permeation	Results, Discussion

Derivation of the composite counts. The figures quoted in the main text were obtained as follows, in each case on the Web of Science export restricted to document types Article and Review:

- *"one of 567 addressed epidemiological cutoff values"*: S1 after document-type restriction (n = 567); records matching ECOFF or epidemiological cut*.
- *"three mentioned a cure endpoint together with MIC determination"*: S1; records matching (clinical OR mycological) adjacent to (cure OR outcome OR response) AND (MIC OR minimum inhibitory).
- *"25 of 383 biofilm records used polymicrobial designs"*: S2 (n = 1,125); denominator, records matching biofilm*; numerator, those additionally matching the polymicrobial term set.
- *"13 records combine an explicit concentration scale with both commensal and host-cell endpoints"*: S4 (n = 1,208); intersection of the concentration-scale, commensal, and host-cell term sets. Of these, records additionally matching vagin* or vulvo* were counted as vaginal (n = 2).
- *"175 botanical records; 68 with quantitative activity, 54 formulation, 38 biofilm, 26 epithelial, 18 commensal, 5 with both"*: population block export (n = 4,814); denominator, records matching the plant-derived term set; numerators, intersections with the corresponding domain sets.
- "six primary studies testing a plant-derived preparation against both Candida and lactobacilli": derived by full-text reading of the 18 records in the population block matching both the plant-derived and commensal term sets, not by term matching alone. Classification of each study by domain, concentration series, and species panel is given in Table S2.

Stated limitations of the method. Term matching identifies mention, not experimental testing, so every count is an upper bound on the number of studies that addressed the domain. Keywords Plus is machine-generated from cited references and inflates topic-field counts; the corresponding block counts are reported above. The plant-derived subset was extracted automatically by the term set above, with no human selection step; the sensitivity of that term set was tested by dual independent screening after peer review and the subset was extended accordingly (S1.11). Domain coding of the plant-derived subset was performed in duplicate, as set

out in S1.8; full-text recoding in the validation substudy and eligibility judgements for the narrative synthesis were single-reviewer tasks. **Counts derive from Web of Science alone, for the field-structure reasons stated in the Methods; PubMed and Scopus were used for identification of literature and for the cross-database overlap analysis, and were not used for primary mapping enumeration; Scopus was subsequently analysed separately in the prespecified cross-corpus sensitivity analysis.**

S1.8. Dual coding and adjudication rules

All 175 records in the botanical subset were coded independently by two reviewers, working from title, abstract, and keyword fields without access to each other's coding, using the term sets and definitions in the Results and Discussion. Agreement before reconciliation, by Cohen's kappa with 95% confidence intervals:

Domain	Percent agreement	Cohen's kappa	95% CI
Quantitative antifungal activity	88.0	0.75	0.65 to 0.85
Formulation	93.7	0.86	0.78 to 0.94
Biofilm	98.9	0.96	0.90 to 1.00
Host-cell or epithelial endpoint	95.4	0.80	0.66 to 0.94
Commensal endpoint	100.0	1.00	1.00

The 42 discordant record-domain pairs were reconciled by discussion. Four boundary conditions emerged during reconciliation that the initial coding manual had not specified, and each was resolved by rule and then applied to every affected record:

Rule 1, in vivo dosing. A dose expressed per unit body weight, such as milligrams per kilogram in a murine model, was not accepted as a quantified antifungal concentration. The value is an administered dose rather than a concentration to which the organism is exposed, and it cannot be placed on the exposure axis that the selectivity window requires. Studies reporting only such values were coded as not providing quantitative antifungal activity, irrespective of the biological result obtained.

Rule 2, animal models and host compatibility. For the same reason, survival or histological outcomes in animal models, including murine, *Galleria mellonella*, and *Caenorhabditis elegans* systems, were not counted as host-cell compatibility. That domain was reserved for measurements on cells or reconstructed tissue producing a concentration-referenced endpoint. In vivo progression is captured separately as a qualitative descriptor in Table 1.

Rule 3, formulation. A delivery system was counted only where it was developed for vaginal administration. Materials and devices not intended for that route, such as a nanofibrous membrane for a sanitary product, were excluded.

Rule 4, direction of the intervention. Where lactobacilli constituted part of the intervention rather than the organism whose survival was assessed, the record was coded as not testing

commensal compatibility. This distinction accounts for the largest single group of discordant records in that domain.

Consensus counts, on which all figures in the main text are based:

Domain	Reviewer 1	Reviewer 2	Consensus	Automated screen
Quantitative antifungal activity	58	77	64	68
Formulation	57	66	59	54
Biofilm	28	26	27	38
Host-cell or epithelial endpoint	22	24	25	27
Commensal endpoint	8	8	8	18
Host and commensal together	n/a	n/a	3	6

The comparison between the automated screen and the consensus is itself informative. For the commensal domain, automated term matching identified more than twice the number of records that independent reading confirmed as having exposed lactobacilli to the test compound and measured an outcome. This is the clearest available estimate of the divergence between mention and measurement in this literature, and it applies in the same direction to every count derived by term matching alone elsewhere in this review.

Sensitivity of the consensus to the adjudication rules. In nine records the two reviewers agreed on a code that the adjudicated consensus does not carry: four in the quantitative antifungal domain and five in the formulation domain. In each case the adjudication applied one of the boundary rules above, most often the exclusion of in vivo dosing as a quantified concentration or the restriction of formulation to preparations intended for vaginal administration, and decided against both abstract-level readings. The adjudicated codes were retained, on the grounds that a rule applied deliberately is not overturned by two readers who were more permissive than the rule. Had the consensus instead followed reviewer agreement in those nine records, quantitative antifungal activity would rise from 64 to 68 of 155 primary studies (41.3% to 43.9%) and formulation from 59 to 62 (38.1% to 40.0%); biofilm, host-cell, and commensal counts are unchanged, the ordering of domains is unchanged, and Cochran's Q rises from 79.49 to 89.76. The conclusions do not depend on this choice.

Added block (revision). The 146 records added after the term-filter check (S1.11) were coded by the same two reviewers under the same manual, with the four rules above applied prospectively. Agreement before reconciliation: quantitative antifungal activity 98.6%, kappa 0.97 (95% CI 0.92 to 1.00); formulation 93.8%, kappa 0.87 (0.78 to 0.95); biofilm 98.6%, kappa 0.95 (0.89 to 1.00); host-cell or epithelial endpoint 95.9%, kappa 0.79 (0.63 to 0.95); commensal endpoint 97.9%, kappa 0.76 (0.49 to 1.00); 22 discordant record-domain pairs were reconciled. Consensus counts among the 138 added primary studies: quantitative antifungal activity 41 (29.7%), formulation 49 (35.5%), biofilm 26 (18.8%), host-cell 16 (11.6%), commensal 5 (3.6%), host and commensal together 0. Across the whole corpus of 321 records kappa was 0.84, 0.86, 0.96, 0.80, and 0.89 for the five domains (64 discordant pairs), and among the 293 primary studies the consensus counts were 105 (35.8%), 108 (36.9%), 53 (18.1%), 41 (14.0%), and 13 (4.4%), with 3 (1.0%) reporting host-cell and commensal endpoints together; Cochran's $Q = 132.8$ ($df = 4$, $p < 0.0001$), and the ordering of domains was unchanged (analysis_S1_extended.py in Data S1).

S1.9. Reporting standard and PRISMA-ScR checklist

This study is an evidence map with a derived conceptual framework, not a systematic review of intervention effects. Reporting follows the PRISMA extension for scoping reviews (PRISMA-ScR) for those items that apply to an evidence map. Three departures are material and are listed against the relevant items below, as well as in Section 5 of the main text: the absence of a prospectively registered protocol, single-reviewer screening and eligibility assessment, and the absence of a validated risk-of-bias instrument. The checklist is provided so that these departures can be located rather than inferred.

Item	Checklist item	Reported in, or departure
1	Title	Title page. The report is identified as a translational framework derived from an evidence map rather than as a systematic review.
2	Structured summary	Abstract. States databases, the coded corpus, the coding strategy, the validation substudy, and the hypothesis-generating status of the framework.
3	Rationale	Introduction, paragraphs 1 to 6.
4	Objectives	Introduction, final three paragraphs. The primary question and the two components of the design are stated explicitly.

Item	Checklist item	Reported in, or departure
5	Protocol and registration	Section 2.1. Departure: no protocol was prepared in advance and the study was not registered. Four coding boundary rules were adopted during reconciliation rather than prespecified, and are reported with the number of records affected in S1.8.
6	Eligibility criteria	Section 2.3 and S1.5.
7	Information sources	Section 2.2 and S1.1.
8	Search	Section 2.2, S1.2, S1.3, and S1.4. Departure: the concept blocks, the five searches, the database-specific translation rules, and two invalidated runs are reported in full, but the complete query strings as executed are not reproduced verbatim for every database.
9	Selection of sources of evidence	Section 2.3, Figure 1, and S1.1. The analytic subset was extracted automatically by a predefined term set, so there was no human selection step to duplicate. The sensitivity of the term set was assessed after peer review by dual independent screening of 484 records (kappa 0.96; S1.11) and the subset was extended (Section 2.3.1). Full-text recoding in the validation substudy and eligibility judgements for the narrative synthesis were single-reviewer tasks, stated as a limitation in Section 5.
10	Data charting process	Sections 2.4 to 2.6, S1.7, and S1.8. Charting was performed independently by two reviewers on the analytic subset and reconciled to consensus.
11	Data items	Section 2.4 and S1.7. Five domains, with the term sets and decision rules reproduced in full.

Item	Checklist item	Reported in, or departure
12	Critical appraisal of individual sources of evidence	Section 2.8 and S2. Departure: no validated risk-of-bias instrument was applied. Appraisal is domain-coverage based and, for the commensal subset, study-level against the three domains of the proposed framework.
13	Synthesis of results	Section 2.7. Wilson intervals, Cochran's Q, exact pairwise McNemar tests with Holm adjustment, and exact binomial intervals for the validation substudy. Departure: inference was confined to the Web of Science corpus, as stated in Section 2.7 and Section 5.
14	Selection of sources of evidence	Section 3.1 and Figure 1.
15	Characteristics of sources of evidence	Sections 3.4, 3.7, and 3.8; S2.
16	Critical appraisal within sources of evidence	Sections 3.7 and 3.8; S2. Reported as evidence coverage and concentration-range adequacy rather than as a bias score.
17	Results of individual sources of evidence	Sections 3.7, 4.7, and S2; Table 1 of the main text.
18	Synthesis of results	Sections 3.4 to 3.6, Figures 2 and 3, and Data S1.
19	Summary of evidence	Sections 4.1 and 6.
20	Limitations	Section 5, items one to eleven.
21	Conclusions	Section 6. The distinction between what the mapping demonstrates and what the framework proposes is stated explicitly.
22	Funding	Funding statement in the main text.

Items 12 and 16 are optional within PRISMA-ScR. They are addressed here in the form appropriate to the object of the mapping, which is the coverage and concentration-range adequacy of reported evidence rather than the internal validity of individual effect estimates.

Item 9 of the checklist requires a flow of sources of evidence. That flow is given as Figure 1 of the main text, drawn in the PRISMA-ScR band structure, with the reason and count stated for every exclusion at each stage. It is not duplicated here.

S1.10. Prespecified protocol for the cross-database sensitivity analysis

Domain-level inference in this study was confined to the Web of Science corpus for the field-structure reasons given in Section 2.7, and that was a design decision rather than a prespecified hypothesis. Because the cross-database overlap analysis in S1.3 shows that no single database contained more than approximately two-thirds of the pooled set, the generality of the domain ordering cannot be assumed. The protocol below was specified in advance of the sensitivity analysis itself, not in advance of the whole study, so that the analysis could be executed without further analytical discretion once the primary mapping was complete. It has since been executed in part, and the result is reported in Section 3.4.1 of the main text. What was executed corresponds to steps 1, 3, 4, and 5 for the Scopus corpus: the frozen term dictionary was applied by the same automated routine, and the prespecified outcome, the ordering of the five domains, was preserved. Two departures from the protocol should be noted. The consensus reading of primary studies specified in step 3 was not performed, so the comparison is between automated term matching in each corpus rather than between consensus-coded sets, and the denominators are all retrieved records rather than primary studies. PubMed could not be included: the available export contained titles only, without abstracts or keyword fields, so the routine had nothing to interrogate in four of its five fields. The comparator used for Web of Science is the automated-screen column already reported in S1.8, which makes the comparison reproducible from data published with this article.

Step	Procedure	Prespecified decision rule
1. Corpus construction	Re-execute the plant-derived population search in PubMed and in Scopus using the strings already reported in S1.3 and S1.4, on a single day, and export title, abstract, and all available keyword fields.	Records are retained irrespective of whether they appear in the Web of Science set. No deduplication against the Web of Science corpus is performed, since the object is the behaviour of each corpus separately.
2. Field harmonization	Concatenate title, abstract, author keywords, and, where available, indexer-assigned keywords. Record for each corpus which fields were present.	Where a corpus lacks indexer keywords, the analysis is run twice for the Web of Science corpus as well, once with and once without Keywords Plus, so that the comparison is between like fields rather than between databases.
3. Coding	Apply the coding dictionary in S1.7 unchanged, by the same automated routine, followed by consensus reading of the same five domains in the primary studies.	The dictionary is frozen. Any term added during this analysis invalidates the comparison and must be reported as a separate exploratory analysis.
4. Primary outcome	For each corpus, compute domain prevalence on primary studies with Wilson intervals, Cochran's Q, and pairwise exact McNemar tests with Holm adjustment.	The prespecified outcome is the ordering of the five domains, not the individual percentages.

Step	Procedure	Prespecified decision rule
5. Interpretation	Compare the domain ordering across the three corpora.	The finding is considered robust if commensal coverage is the lowest domain and is separated from host-cell coverage in each corpus. If the ordering is not preserved, the main-text claim must be restricted to the Web of Science corpus, and the difference between corpora becomes the reportable result.

Results of the cross-corpus sensitivity analysis (moved from Section 3.4.1 of the first submitted version)

The coding routine and term dictionary were applied without modification to the plant-derived subsets retrieved independently from Scopus (n = 316) and PubMed (n = 251), following the protocol prespecified in S1.10, with the ordering of domains as the prespecified outcome. The retrieval routes are not identical and the comparison is not a controlled one: the Web of Science subset was obtained by applying the botanical term set to the population block, whereas the Scopus and PubMed subsets are the corresponding combined search (S3 in Table S1) executed in those databases. Denominators are all retrieved records in each corpus rather than the primary-study denominator used in Section 3.4, since term matching does not distinguish document type.

In Scopus the ordering was identical to that obtained in Web of Science: quantitative antifungal activity (41.1%), formulation (26.9%), biofilm (18.0%), host-cell endpoints (16.1%), and commensal testing (13.0%). The corresponding Web of Science values by the same routine were 38.9%, 30.9%, 21.7%, 15.4%, and 10.3%; these are the automated-screen counts on the 175-record subset already reported in S1.8, so the comparator is reproducible from the published supplementary data rather than generated for this analysis. Domain coverage differed significantly within each corpus (Cochran's Q = 50.7 for Web of Science and 95.3 for Scopus, df = 4, both $p < 0.001$). No formal cross-corpus test of proportion differences or equivalence was performed; the prespecified sensitivity outcome was preservation of the domain ordering, which was observed in Scopus. What the analysis supports is the prespecified outcome, that the ordering of domains is preserved when the analytic corpus is changed.

The internal consistency of the routine is worth stating. Applied to the Web of Science subset, term matching returned commensal testing in 18 of 175 records (10.3%), against 8 of the same 175 records (4.6%) by consensus coding, a ratio of 2.25. Both figures are on the same denominator and both are already reported in Section 3.4 and S1.8. The ratio reproduces independently the overestimation factor documented for this domain, and is the strongest available indication that the routine behaved in the sensitivity analysis exactly as it behaved in the primary analysis.

PubMed could not be included on comparable terms, and the reason is the one given in Section 2.7 rather than a property of the database. The available export contained titles only, without abstracts or keyword fields, while the routine interrogates title, abstract, and both keyword fields. The effect is visible and diagnostic: quantitative antifungal activity was returned in 0.4% of PubMed records,

since inhibitory concentrations are almost never stated in a title, and the ordering was consequently disrupted. This is a difference in available fields rather than in literature, and it is the same field-structure argument that motivated the choice of analytic corpus.

S1.11. Term-filter sensitivity check and extension of the plant-derived subset

The 14-term plant-derived filter of S1.7 was constructed around essential-oil and terpenoid chemistry. After peer review its sensitivity was tested as follows; every step is executed by screening_sweep_S1.py in Data S1 with the retained seed 20260911. Step 1: an expanded set of 35 term groups was applied to the 4,639 Article and Review records the original filter had excluded: herbal, herbs, polyherbal; plant, plants; extract, extracts; phyto*; natural product, natural compound, natural substance, natural antifungal, natural remedy; propolis; flavon*, polyphenol, phenolic, tannin, saponin, alkaloid, glycoside, coumarin, stilbene, lignan, resveratrol, quercetin, catechin, berberine; allicin, garlic, Allium; cinnam*; Origanum, oregano; Lavandula, lavender; Mentha, mint, menthol; Ocimum, basil; Thymus vulgaris, Thymus zygis, Thymus capitatus, thyme; neem, Azadirachta; Aloe; Nigella, black seed, black cumin; clove, Syzygium; Zingiber, ginger, turmeric, Curcuma; green tea, Camellia; lemongrass, Cymbopogon, citral; limonene, linalool, cineole, eucalyptol, pinene; Rosmarinus, rosemary, Salvia, sage; chamomile, Calendula, Matricaria; Eucalyptus; Zataria, Satureja, Thymbra; Punica, pomegranate, cranberry, Vaccinium; Pelargonium, geranium; Hypericum, Artemisia, Achillea; Ayurved*, traditional (Chinese or herbal) medicine, TCM, Unani, Kampo; decoction, tincture; coconut oil, olive oil, seed oil. It flagged 308 records. Step 2: those 308 records and a random control sample of 150 records flagged by no term were shuffled and screened independently by two evaluators, blind to stratum and to each other, against one question: does the record report, test, or evaluate a plant-derived preparation or compound (whole-plant extract, essential oil, isolated phytochemical, herbal or polyherbal product, traditional plant-based formulation) in a vulvovaginal candidiasis context, in any study design? Allowed answers were yes, no, bee product, and unsure. Decision rules applied at reconciliation: (a) plant material used only as a vehicle, base, or excipient is not counted; (b) synthetic or semi-synthetic derivatives of a natural product are not counted unless the tested compound is itself the plant constituent; (c) marine algae are not plants for this corpus; (d) a named natural compound whose plant origin is not stated in the record is resolved from the full text; (e) a vulvovaginal context means vaginal isolates, a vaginal or vulvovaginal model, or women with the disease. Step 3: a further sweep with named phytochemicals and plant genera (for example lawsone, hypericin, perillaldehyde, shikonin, ajoene, glycyrrhetinic acid, Centella) was applied to the 4,181 records still unscreened, and its 26 candidates were screened by the same two evaluators.

Results. In step 2 the evaluators agreed on 448 of 458 records (97.8%); Cohen's kappa was 0.96 (95% CI 0.93 to 0.98) over the four answer categories and 0.97 (0.94 to 0.99) for yes against any other answer. By consensus, 126 of the 308 flagged records and 2 of the 150 controls (lawsone, hypericin) were plant-derived studies in a vulvovaginal candidiasis context; 20 records concerned propolis, geopropolis, or honey and were set aside as bee products, which are not included in the plant-derived subset and are reported here for completeness; 10 discordant records were resolved to 4 yes, 5 no, and 1 bee product, and 7 records marked unsure by both evaluators to 2 yes, 4 no, and 1 still unresolved for want of full text (a 2001 report on a natural product for vulvovaginitis whose composition could not be established). In step 3 the evaluators agreed on all 26 candidates (kappa 1.00) and confirmed 13; at a final consistency check one of these was excluded under rule (a), because the plant material (sesame oil) was the vehicle of a fungal

polysaccharide, and a bacterial vaginosis case series of a Centella asiatica gel remained excluded under rule (e) although the vulvovaginal candidiasis report of the same gel was included. The extension therefore added 146 records (138 primary studies and 8 reviews), and the original filter captured 175 of the 321 records of the final corpus (54.5%). Among records matched by no term, the residual false-negative rate is estimated at 2 of 150 (1.3%, exact 95% CI 0.2 to 4.7%), corresponding to roughly 7 to 200 records among the 4,181 records not screened by hand; it is reported as a limitation. All confirmed records were coded for the five domains by the duplicate procedure of S1.8, with the four boundary rules applied prospectively; the screening workbooks, the reconciliation record, and the coding of the added block are supplied in Data S1, and agreement for the added block is reported in S1.8.

Table S2. Appraisal of primary plant-derived studies testing both Candida and vaginal commensals

S2.1. Scope and derivation

Of 175 records in the Web of Science population block matching plant-derived terms, 18 also matched commensal terminology on the automated screen. Independent reading reduced these to 8 in which lactobacilli were actually exposed to the test compound and an endpoint was measured; the other 10 were reviews, practitioner or patient surveys, or primary reports in which commensals were mentioned without being tested. Of those 8, five are primary studies in which a plant-derived preparation was tested against both *Candida* and lactobacilli under conditions permitting comparison on a shared concentration axis. One further qualifying study was identified through the Scopus set and is included, giving *six comparative studies in total*. Each was appraised against the three domains of the proposed selectivity window and against the concentration ranges over which each domain was examined.

S2.2. Appraisal

Study	*Candida* tested	Commensal tested	Host cell tested	Concentration design	Ranges overlap the antifungally active concentration	All three domains
Di Vito et al. 2015 [23]	20 strains, vaginal and reference, including azole-resistant	*L. casei*, *L. acidophilus*, *L. plantarum*, *Bifidobacterium animalis*	Not tested	Graded series, 0.0078 to 4% v/v	Yes, for the two domains tested	No
Bezerra et al. 2025 [24]	*C. parapsilosis*, *C. glabrata*, *C. tropicalis* reference strains	*L. jensenii* ATCC 55920	VK2/E6E7 vaginal epithelial line	Graded series for all three domains	Yes	**Yes**

Study	*Candida* tested	Commensal tested	Host cell tested	Concentration design	Ranges overlap the antifungally active concentration	All three domains
Fernandes et al. 2025 [25]	3 drug-resistant clinical isolates	*L. gasseri* ATCC 33323	Reconstituted human vaginal epithelium, LDH release	Single fixed exposure, vapour phase	Not applicable, single point	Domains present, no gradient
Park et al. 2025 [26]	*C. albicans* KCTC 7965	*L. crispatus* KCTC 5054	Human dermal fibroblasts, HeLa	Graded series for all three domains	No; host cells tested to 100 µg/mL against a *Candida* MIC of 500 µg/mL	No
Iseppi et al. 2020 [27]	2 vaginal clinical isolates and 1 reference strain	4 unspeciated vaginal *Lactobacillus* isolates	Not tested	Graded series, both organisms	Yes, for the two domains tested	No
Saracino et al. 2022 [28]	18 isolates of vaginal origin	*L. acidophilus* La-14	Not tested; GRAS status and published oral and dermal no-effect levels cited instead	Graded series, both organisms	Yes, for the two domains tested	No

S2.3. Species panel and its adequacy

Study	Commensal species used	Dominant in healthy vaginal communities
Di Vito et al. 2015	*L. casei*, *L. acidophilus*, *L. plantarum*, *B. animalis*	No
Bezerra et al. 2025	*L. jensenii*	Yes
Fernandes et al. 2025	*L. gasseri*	Yes
Park et al. 2025	*L. crispatus*	Yes
Iseppi et al. 2020	Unspeciated vaginal isolates	Undetermined
Saracino et al. 2022	*L. acidophilus*	No

Three of the six studies used a species that dominates healthy vaginal communities. Where the protected organism is not vaginally dominant, a selectivity margin measured against it is a real measurement against an organism whose displacement does not correspond to the ecological outcome of concern.

S2.4. Reported selectivity margins

Values are reproduced as reported by the original authors and are not recalculated to a common basis. They derive from different assays, endpoints, and units, and are not directly comparable.

Study	Compound	Margin as reported
Di Vito et al. 2015	Tea tree oil	*Candida* MIC 0.25% v/v; *Lactobacillus* MIC 2 to 4% v/v; *B. animalis* MIC 0.5% v/v
Iseppi et al. 2020	*Lavandula x intermedia* oil	*Candida* MIC 9 to 18 μg/mL; lactobacilli 72 to 144 μg/mL
Iseppi et al. 2020	*Mentha arvensis* oil	Lactobacilli 9 to 18 μg/mL; *Candida* 18 to 144 μg/mL, ordering reversed
Saracino et al. 2022	Cinnamaldehyde, α-pinene, eugenol	Commensal MIC approximately 5.5 to 6.6 times the mean *Candida* MIC
Bezerra et al. 2025	Miconazole	Epithelial cytotoxicity from 5.94 μg/mL; *L. jensenii* viability reduced above 95 μg/mL
Park et al. 2025	Basil essential oil	*L. crispatus* viability above 95% to 2000 μg/mL against a *Candida* MIC of 500 μg/mL; host cells not tested at the active concentration
Fernandes et al. 2025	White thyme oil, vapour phase	*L. gasseri* unaffected during co-colonization at the single exposure tested

S2.5. Worked application of the operational definition

The operational definition proposed in Section 4.8.1 and Table 2 of the main text and Figure S1 of this file is applied below to the six studies appraised in this table, to demonstrate that it can be computed and to show what it returns. All values are tier 1 nominal concentrations reproduced as reported by the original authors and not recalculated to a common basis; they derive from different assays, endpoints, and units, and the resulting ratios are ranking quantities only. CF, CE, and CM are as defined in the main text.

Study and compound	Values as reported (tier 1, nominal)	Result under the operational definition

Study and compound	Values as reported (tier 1, nominal)	Result under the operational definition
Di Vito et al. 2015, tea tree oil [23]	CF, *Candida* MIC 0.25% v/v. CM, lactobacilli 2 to 4% v/v and *B. animalis* 0.5% v/v. CE not tested.	S undefined; the epithelial domain was not measured. Microbiota selectivity ratio 2 against the most susceptible protected organism and 8 to 16 against the lactobacilli alone, which is the eightfold difference produced by the choice of protected organism. The panel contains no species dominant in healthy vaginal communities.
Bezerra et al. 2025, miconazole in a ureasil-polyether ovule [24]	CF 1.25 (*C. tropicalis*), 2.5 (*C. parapsilosis*), and 12.5 μg/mL (*C. glabrata*). CE 5.94 μg/mL. CM above 95 μg/mL.	S computable, and species-dependent: approximately 4.8 and 2.4 for the first two species, so the window is open and constrained by the epithelium in both; approximately 0.5 for *C. glabrata*, so the window is closed. The microbiota selectivity ratio exceeds 7 in every case, which is the value a programme testing only lactobacilli would have reported.
Fernandes et al. 2025, white thyme oil vapour [25]	Single fixed exposure in a closed chamber; fungal activity by inhibition zone diameter. All three domains present.	S undefined for all three domains: no concentration series, so no bound can be located. This is the case in which selectivity is demonstrated at a point and the interval remains unknown.
Park et al. 2025, basil essential oil [26]	CF 500 μg/mL. CM not reached at 2000 μg/mL. CE tested only to 100 μg/mL, with viability already about 73% at that limit.	S undefined under the second rule: the epithelial range does not span CF, so the domain was not tested at the concentration that matters. Microbiota selectivity ratio greater than 4, reported as a lower bound.
Iseppi et al. 2020, *Lavandula x intermedia* oil [27]	CF 9 to 18 μg/mL. CM 72 to 144 μg/mL. CE not tested.	S undefined; the epithelial domain was not measured. Microbiota selectivity ratio approximately 8.

Study and compound	Values as reported (tier 1, nominal)	Result under the operational definition
Iseppi et al. 2020, *Mentha arvensis* oil [27]	CM 9 to 18 µg/mL. CF 18 to 144 µg/mL. CE not tested.	Window closed on the commensal axis under the tested conditions: the commensal threshold lies at or below the fungal effective concentration. Reported as closed, not as a negative value; reformulation or combination would require redetermination of the relevant endpoints.
Saracino et al. 2022, cinnamaldehyde, α-pinene, eugenol [28]	Commensal MIC approximately 5.5 to 6.6 times the mean *Candida* MIC. CE not tested; regulatory no-effect levels for oral and dermal exposure cited instead.	S undefined; the epithelial domain was not measured, and a regulatory designation for another route is not a substitute under the reporting requirements of Table 2.

The selectivity index is computable in one of the six studies. In that study it is open for two Candida species and closed for a third, and in every species the constraining domain is the epithelium rather than the microbiota. In one further study the window is closed on the commensal axis. In the remaining four the index is undefined, in three cases because the epithelial domain was not measured and in one because no concentration series was used. This distribution is the reason the definition specifies an undefined result rather than permitting a ratio to be computed from the domains that happen to have been measured: four of these seven study-compound entries, seven rather than six because one study contributed two oils, would otherwise yield a favourable-looking number generated by an untested domain.

S2.6. Scoring rules applied in Table 1 of the main text

●●● evidence from multiple independent studies including clinically relevant isolates or a formulation-specific model; ●● evidence from at least one primary study under conditions relevant to vaginal exposure; ● preliminary or single-study evidence; empty cell, domain not investigated under relevant conditions. An empty cell is never interpreted as a negative result and never as evidence of safety.

Supplementary Note S3. Laboratory interpretive practice for vaginal Candida isolates

This note contains the material on laboratory practice that formed Sections 4.3.1 to 4.3.3 and Tables 1 and 2 of the first submitted version of the main text. It was moved here at the reviewers' request because it is background to the argument rather than a result of the evidence map or a component of the proposed framework. Section 4.3 of the main text retains only the points that bear on the framework.

S3.1. When should the laboratory escalate testing?

Routine expanded AFST is generally unnecessary in uncomplicated VVC. The laboratory role becomes important when symptoms persist after an appropriate course of treatment, when VVC is recurrent, when cultures remain positive during suppressive therapy, after repeated azole exposure, in clinically relevant immunosuppression, and when non-albicans Candida or unusual species are recovered [1,4,5]. CDC guidance specifically recommends culture or molecular confirmation in complicated VVC and consideration of susceptibility testing in symptomatic patients who remain culture-positive despite maintenance therapy [1].

Species-level identification should precede any expansion of the antifungal panel. Candida albicans, Nakaseomyces glabratus, Pichia kudriavzevii, Candida tropicalis, and the Candida parapsilosis complex differ in baseline susceptibility patterns, in intrinsic resistance, and in mechanisms of acquired resistance [35,36]. Identification alone therefore resolves a proportion of apparent treatment failures without any susceptibility testing at all.

Treatment failure should not be equated with microbiological resistance. Poor adherence, insufficient duration, formulation-related exposure, diagnostic error including non-candidal vulvovaginal disease, biofilm, host factors, reinfection or recolonization, and ecological disturbance can all produce persistent symptoms in the presence of a fully susceptible isolate. A susceptibility result cannot distinguish between these possibilities, which is precisely why indiscriminate panels generate false reassurance in one direction and unwarranted therapeutic escalation in the other.

S3.2. Commercial systems: quantitative MIC versus fixed-concentration phenotype

Commercial systems illustrate why analytical output must be distinguished from clinical interpretation. MICRONAUT-AM is a colorimetric broth microdilution system designed to generate quantitative MICs for a panel of antifungal agents and to support EUCAST-oriented interpretation, and has been evaluated as such against a comparator commercial method [38]. In 2026, EUCAST published specific guidance on qualifying the in-house performance of commercial antifungal susceptibility tests before adopting EUCAST breakpoints [39]. This guidance is directly relevant to routine laboratories: a commercial MIC can be clinically useful, but local performance and the applicability of the breakpoint framework must be demonstrated before categorical results are issued.

Commercial MIC values are also method dependent. In a comparison of Sensititre YeastOne and MICRONAUT-AM, Philips et al. demonstrated significant method-associated variability, with essential agreement of at least 90% achieved only for selected agents and systematic differences in MIC distributions between systems [38]. Numeric MIC output does not make different commercial systems analytically interchangeable, and this matters for any attempt to pool topical MIC data across centres.

Integral System YEASTS Plus represents a different paradigm, combining identification with fixed-concentration phenotypic testing of a broader set of antifungals including several local agents; an evaluation against CLSI broth microdilution reported poor susceptibility test performance and substantial identification disagreement [40]. More recent work continues to use it for descriptive profiling [41], including a series of 300 genital yeast isolates from 4,569 symptomatic women in which the panel screened every strain and 43 flagged isolates were re-tested by broth microdilution [42]. Screen-then-confirm is the defensible way to use a fixed-concentration panel, but it has a direction: confirmation is applied only to flagged isolates, so the resulting MIC distributions are conditioned on the screen and describe the flagged subset rather than the collection. In that series 94.7 per cent of the selected C. albicans isolates were resistant to fluconazole, whereas the same isolates represent 12.0 per cent of the 150 C. albicans strains examined. Both figures are correctly reported, and the distance between them is the point. Such a design supports statements about how often a flagged phenotype is confirmed, not about the MIC distribution of a population, which is what the surveillance role proposed here requires.

S3.3. Three reporting tiers

A practical laboratory response can be organized into three reporting levels: clinically interpreted AFST when a valid breakpoint exists; quantitative activity without categorical interpretation when a topical MIC is generated for epidemiological or investigational reasons; and research-only endpoints for botanical compounds and experimental combinations. The corresponding terminology, permitted conclusions, and conclusions to avoid are set out in Table S3. Adopting this vocabulary requires no new susceptibility category and no change to accredited methods; it changes only what the laboratory is willing to assert.

S3.4. Conventional topical agents as comparators

This subsection reproduces the comparator discussion of Section 4.4 of the first submitted version, compressed in the main text at the reviewers' request.

Clotrimazole, miconazole, econazole, and fenticonazole remain clinically established local antifungals, but widespread clinical use at the population level does not generate individual MIC breakpoints for vaginal treatment. Their immediate research value lies elsewhere: in species-specific MIC distributions, in detection of unusual phenotypes, and in prospective correlation with outcome. The association between fluconazole non-wild-type phenotypes and higher MICs to several other azoles reported in vaginal isolates supports investigation of cross-azole susceptibility patterns, but does not establish universal cross-resistance and

should not be used to withhold a topical imidazole on the basis of a fluconazole result alone [17,35].

One historical dataset illustrates the point of principle. Tioconazole and fluconazole were tested against the same 32 yeast isolates by a single method, with a sixteenfold difference in MIC50, at 0.5 mg/L or below for tioconazole against 8 mg/L for fluconazole [21]. The values predate current reference methodology and were generated on isolates of bloodstream origin, so they carry no interpretive weight for vaginal disease, but they show that a fluconazole result is a poor proxy for the activity of a topical imidazole even in vitro, before any question of achieved local concentration arises.

Recent practice shows that the interpretive vacuum is filled informally rather than left empty. In one series of genital yeast isolates, categorical resistance to clotrimazole was assigned above a MIC of 1 mg/L on the authority of an earlier publication, and nystatin results were read against a limit the authors record as suggested by other investigators rather than defined by CLSI or EUCAST [42]. The thresholds are explicitly attributed, so this is not a criticism of the study but an illustration of the mechanism: where no validated criterion exists, an informal one is adopted because a categorical answer is expected, and it then circulates as though it were interpretive. The same series defined the MIC endpoint as complete inhibition for nystatin and fifty per cent for the azoles, so even within one study the two agents are not on one scale.

The imbalance in how these agents have been studied is itself informative. Within the same 567 records, fluconazole is mentioned in 347, clotrimazole in 85, nystatin in 71, and miconazole in 64, but econazole in 7 and fenticonazole in 5. Several clinically established vaginal imidazoles are therefore among the least represented in the susceptibility literature, which is why local MIC distributions for them remain unavailable rather than merely uninterpreted. The mapping describes publication volume and cannot speak to prescribing.

Nystatin is useful because its polyene mechanism provides a mechanistically distinct comparator, so a longitudinal programme could identify patterns in which azole MICs shift while nystatin activity remains stable, or the converse. Such observations would remain descriptive until linked to outcome.

Fluconazole should remain the principal standardized azole comparator wherever an applicable breakpoint exists, not as a surrogate for topical imidazoles but as a clinically interpretable reference phenotype against which topical MIC distributions and botanical activity can be positioned. The analytical value and interpretive limits of each agent are summarized in Table S4.

Table S3. Proposed laboratory terminology for vaginal Candida isolates (Table 1 of the first submitted version).

Evidence available	Recommended term	Permitted conclusion	Conclusion to avoid
Standardized MIC + applicable breakpoint	Clinically interpreted AFST	S/I/R according to current framework	Prediction for another agent or formulation
MIC + established ECOFF, no breakpoint	WT/non-WT phenotype	Population susceptibility phenotype	Clinical resistance
Experimental topical MIC	Quantitative topical antifungal activity	Comparative concentration-dependent activity	Routine vaginal S/I/R
Local ECOFF from a single study	Local epidemiological cutoff	Local distribution description	Universal ECOFF or clinical breakpoint

Fixed-concentration commercial panel

Phenotypic screening result

Growth response under manufacturer conditions

EUCAST-equivalent MIC or S/I/R

Plant-derived compound

Experimental antifungal activity

MIC/MFC/functional effect

Clinical susceptibility

Antifungal + botanical compound

Experimental interaction

Interaction signal under the stated assay

Clinical synergy

Clinical failure

Treatment failure

Need for clinical reassessment

Resistance by definition

Table S4. Antifungal agents relevant to vulvovaginal candidiasis: analytical value and interpretive limits (Table 2 of the first submitted version).

**Agent**	**Class**	**Relevance in VVC**	**Quantitative MIC**	**Topical clinical S/I/R**	**Recommended role**
Fluconazole	Triazole	Oral therapy of VVC and recurrent VVC	Yes; standardized	Not a generic topical breakpoint	Primary clinically interpretable azole comparator
Itraconazole	Triazole				

Selected oral use

Yes

No generic topical extrapolation

Secondary systemic comparator

Voriconazole

Triazole

Selected refractory disease

Yes

No

Specialist AFST

Amphotericin B

Polyene

Compounded local use in refractory disease

Yes

No general topical breakpoint

Specialist comparator

Flucytosine

Antimetabolite

Compounded local use in refractory disease

Yes

No general topical breakpoint

Specialist comparator

Anidulafungin / micafungin

Echinocandins

Systemic candidiasis; not routine in VVC

Yes

Not relevant to routine topical therapy

Systemic AFST

Clotrimazole

Imidazole

Major topical VVC agent

Yes, experimentally

No validated topical clinical breakpoint

Topical comparator and MIC surveillance

Miconazole

Imidazole

Major topical VVC agent

Yes, experimentally

No validated topical clinical breakpoint

Topical comparator and formulation studies

Econazole

Imidazole

Topical VVC

Yes, experimentally

No validated topical clinical breakpoint

Epidemiological surveillance

Fenticonazole

Imidazole

Topical VVC

Yes, experimentally

No validated topical clinical breakpoint

Epidemiological surveillance

Nystatin

Polyene

Vaginal therapy, including non-*albicans* disease

Yes, experimentally

No universal validated topical S/R framework

Mechanistically distinct comparator

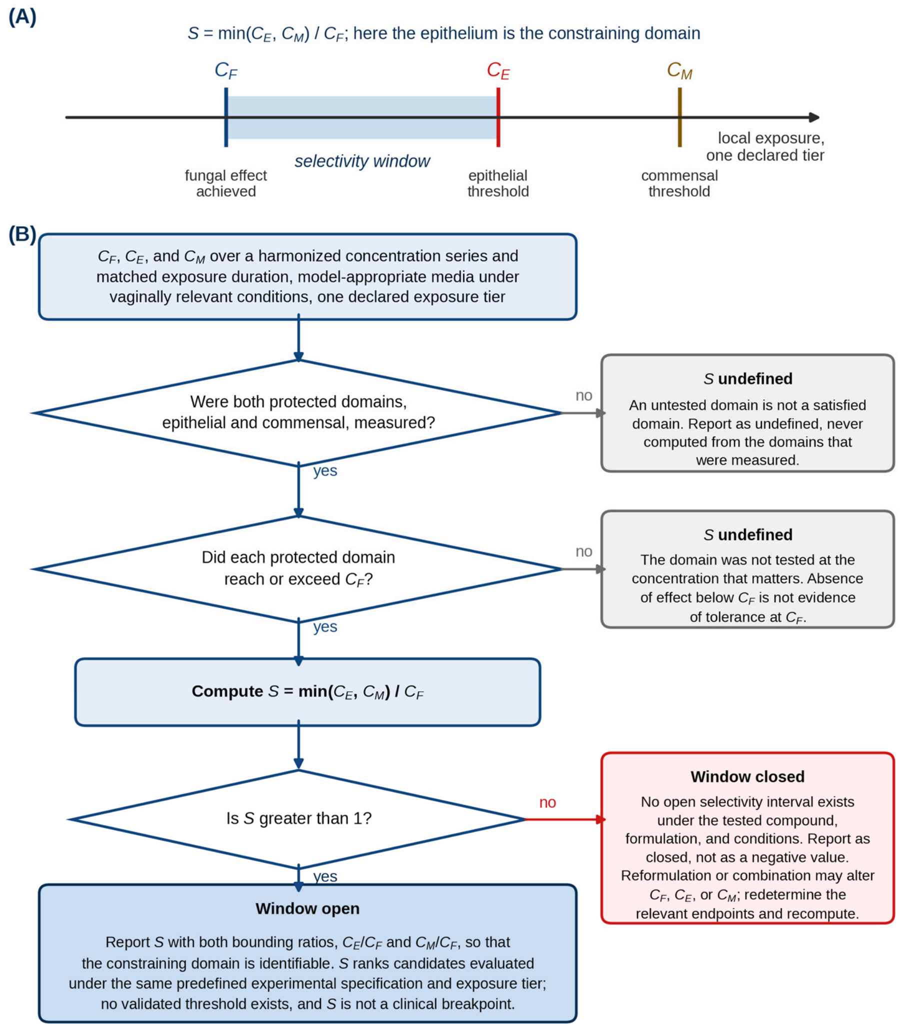

Figure S1. Computing the selectivity index (Figure 6 of the first submitted version). (A) The three concentrations placed on one exposure axis at a single declared tier: the fungal effective concentration CF, the epithelial threshold CE, and the commensal threshold CM. The window is the interval between CF and the smaller of the two protected thresholds, and the index S is the ratio of that smaller upper bound to CF; in the case drawn, the epithelium is the constraining domain. (B) The decision sequence by which S is obtained. Two outcomes return an undefined index rather than a number: a protected domain that was not measured, and a protected domain whose tested range stops below CF. Where S is 1 or less the window is closed and is reported as such, never as a negative value. The index

ranks candidates evaluated under the same predefined experimental specification and exposure tier; no validated threshold value exists, and it is not a clinical breakpoint. A closed result is conditional on the compound, formulation, and conditions tested: reformulation or combination may alter CF, CE, or CM, in which case the relevant endpoints are redetermined and the index recomputed. The metrics admissible for each concentration are specified in Table 2 of the main text.

Supplementary Note S4. Measuring local exposure: the proposed instrument in full

This note reproduces Section 4.10 of the first submitted version, which the main text now summarizes; passages that relied on cross-condition analogies were removed at the reviewers' request.

The most direct way to resolve the topical susceptibility gap is a prospective cohort linking quantitative topical MICs to real treatment exposure and outcome. Such studies are exceptionally scarce: among the 567 susceptibility-related VVC records described in Section 3.2, only three mention a clinical or mycological cure endpoint together with MIC determination, and one is a perspective article rather than a primary cohort [13]. For recurrent or refractory VVC, a minimum dataset should record clinical syndrome and episode number, species identification, prior antifungal exposure, quantitative topical MICs generated within a research protocol, the treatment given with formulation, dose, and duration, clinical and mycological cure, recurrence interval, sequential isolates, and where feasible a cervicovaginal antifungal activity titre. Each element is feasible in a routine service; the value lies in recording them together and prospectively.

Feasibility is supported by existing reference-laboratory programmes: a New Zealand series of 2,345 yeast isolates accumulated between 2001 and 2015 showed how far revised interpretive criteria can change the classification of an unchanged collection [71], which is why isolates should be archived and exposure recorded prospectively. The need is not hypothetical: recent United Kingdom surveillance reports rising rates of non-albicans yeasts and fluconazole resistance among isolates from women with recurrent VVC [72].

One methodological transposition deserves attention. Serum fungicidal concentration testing determines whether a patient’s own serum kills the patient’s own isolate, measuring achieved exposure and biological effect together in the matrix where the drug acts [12]. We identified no cervicovaginal analogue, although a partial precedent exists: both historical tioconazole studies quantified drug in vaginal fluid by bioassay against a laboratory yeast organism [18,21]. That is a drug-concentration assay in the target matrix, not a patient-specific activity measurement, since the organism was a reference strain and the endpoint growth inhibition rather than killing.

We propose a cervicovaginal antifungal activity titre (CV-AAT): cervicovaginal fluid sampled at defined intervals after administration of a topical agent and titrated against the patient's own isolate, with the endpoint defined as inhibition of growth. Where subculture from inhibited wells is added and killing is demonstrated, the corresponding fungicidal

measure may be reported separately as a cervicovaginal fungicidal titre. The distinction matters because most topical azoles are fungistatic at achievable concentrations, and an inhibition-based titre should not be described as fungicidal without the confirmatory step.

CV-AAT is a proposed assay, not an available one, and requires analytical and clinical validation before it can interpret anything: recovery from cervicovaginal fluid, within- and between-run reproducibility, the effect of dilution and of small variable sample volumes, normalization for fluid volume and mucus binding, drug carry-over into subculture, analyte stability, and performance against the patient's own isolate. Only then can the relationship between titre and outcome be tested, which is the question that would justify the assay. We describe the design and its rationale here; we have not performed it.

Feasibility is better documented than its absence suggests. Vaginal fluid volumes of roughly 0.2 to 0.3 mL were obtainable at each sampling interval; one series reported a bioassay error of about 20 per cent, the other that 10.2 per cent of samples were insufficient for assay [18,21]. Substituting the patient's own isolate for the reference organism converts a drug-concentration measurement into an activity measurement, and is the substantive change proposed here.

A nested translational cohort could additionally include biofilm phenotype, epithelial markers, and microbiota composition at baseline and at recurrence. This design would permit direct testing of two questions that are currently answered by assertion: whether a topical MIC predicts outcome, and whether repeated topical azole exposure is associated with measurable shifts in MIC distributions. The second question is more rigorous than assuming that topical azole exposure directly causes systemic azole resistance. Maillard's recent appraisal of whether topical azole use drives antifungal resistance concluded that the evidence base is methodologically heterogeneous across the field as a whole, with much of the resistance signal arising outside the vaginal setting [73]. That conclusion is the strongest available argument for the design proposed here: where the question has been asked across all topical azole use, the answer has been limited by study heterogeneity rather than by absence of interest, and VVC-specific longitudinal data linking exposure to phenotype do not yet exist. Biomarker selection should be tissue-appropriate rather than imported from other settings: mucosal epithelial and inflammatory markers would require their own analytical and clinical qualification before any of them could serve as an outcome.

Prospective validation should also include symptom burden, recurrence, and patient-reported quality of life, because microbiological response does not exhaust the burden of recurrent disease [6], and because help-seeking for intimate symptoms is shaped by stigma; patient-reported and care-seeking variables should be measured rather than assumed.

Within an EUCAST-aligned laboratory, implementation requires no new susceptibility categories. It requires stricter separation of clinically interpretable AFST, quantitative epidemiological activity, and translational testing, supported by the 2026 EUCAST guidance on qualifying commercial antifungal susceptibility tests before adopting EUCAST breakpoints [39]. A two-tier service model is feasible: routine clinical mycology provides

species identification and validated AFST when clinically indicated, while a specialized service archives isolates, generates quantitative topical MIC distributions within a research framework, compares sequential isolates, links exposure to phenotype, and supports translational evaluation of adjuvant formulations. The innovation lies in generating high-quality longitudinal evidence, not in inventing local S/R thresholds.

Reference numbers in this note refer to the reference list of the main text.

Data S1. Record-level dataset, analysis script, and output

S1 dataset

The file coding_dataset.csv contains one row per record in the plant-derived subset (n = 175) with the following columns.

Column	Content
record_id	Anonymous identifier
doi	Digital object identifier, where present
year	Publication year
document_type	primary or review
r1_[domain]	Reviewer 1 coding, five domains
r2_[domain]	Reviewer 2 coding, five domains, performed independently
cons_[domain]	Consensus coding after reconciliation; this is the coding analysed in the main text
validation_sample	Record drawn into the stratified full-text substudy
full_text_available	Full text obtainable for the substudy
ft_[domain]	Coding from the full text, blank where the full text was unavailable

The five domains are antifungal, formulation, biofilm, host, and commensal.

Analysis script

Running `python analysis_S1.py` in the same directory as the dataset reproduces, in order: reviewer agreement with Cohen's kappa and confidence intervals; domain coverage with Wilson intervals on the 155 primary studies and on all 175 records; Cochran's Q; the ten exact pairwise McNemar tests with Holm adjustment; and the full-text validation substudy with exact binomial confidence intervals. The scripts were run with Python 3.13, pandas 3.0, NumPy 2.5, and SciPy 1.18.

"""Supplementary Data S1: reproduces the principal inferential statistics reported for the evidence-mapping and full-text validation analyses.

Input : coding_dataset.csv (record-level coding, n = 175)

```
Usage : python analysis_S1.py
Needs : pandas, numpy, scipy

The dataset contains, for each of the 175 plant-derived records:
  document_type      primary | review
  r1_<domain>        reviewer 1 coding, five domains
  r2_<domain>        reviewer 2 coding, five domains, performed independently
  cons_<domain>      consensus coding after reconciliation; this is what the paper reports
  validation_sample  record was drawn into the stratified full-text substudy
  full_text_available full text obtainable
  ft_<domain>        coding from the full text, blank where unavailable
"""
import pandas as pd, numpy as np, math, json
from scipy import stats

DOM = ['antifungal','formulation','biofilm','host','commensal']
LAB = {'antifungal':'Quantitative antifungal activity','formulation':'Formulation',
       'biofilm':'Biofilm','host':'Host-cell / epithelial','commensal':'Commensal'}

def wilson(k, n, z=1.96):
    if n == 0: return (0.0, 0.0)
    p = k/n; d = 1 + z*z/n
    c = (p + z*z/(2*n))/d
    h = z*math.sqrt(p*(1-p)/n + z*z/(4*n*n))/d
    return (100*max(0, c-h), 100*min(1, c+h))

def cohen_kappa(a, b):
    n = len(a); po = (a == b).mean()
    pa, pb = a.mean(), b.mean()
    pe = pa*pb + (1-pa)*(1-pb)
    k = (po-pe)/(1-pe)
    se = np.sqrt(po*(1-po)/(n*(1-pe)**2))
    return k, po, max(-1.0, k-1.96*se), min(1.0, k+1.96*se)

def cochran_q(X):
    k = X.shape[1]; L = X.sum(1); G = X.sum(0)
    Q = (k-1)*(k*(G**2).sum() - L.sum()**2)/(k*L.sum() - (L**2).sum())
    return Q, k-1, stats.chi2.sf(Q, k-1)

def mcnemar_exact(a, b):
    b01 = int(((a==1)&(b==0)).sum()); b10 = int(((a==0)&(b==1)).sum()); n = b01+b10
    if n == 0: return (b01, b10), 1.0
    p = 2*min(stats.binom.cdf(min(b01, b10), n, 0.5), 0.5)
    return (b01, b10), min(1.0, p)

def holm(ps):
```

```
    idx = np.argsort(ps); out = np.empty(len(ps)); run = 0.0
    for r, i in enumerate(idx):
        v = (len(ps)-r)*ps[i]; run = max(run, v); out[i] = min(1.0, run)
    return out

def fmt_p(p):
    # chi2.sf is used rather than 1 - chi2.cdf, which cancels in the tail;
    # the guard below only applies far beyond double precision.
    return 'p < 1e-300' if p < 1e-300 else f'p = {p:.3g}'

def clopper_pearson(k, n):
    lo = stats.beta.ppf(0.025, k, n-k+1) if k > 0 else 0.0
    hi = stats.beta.ppf(0.975, k+1, n-k) if k < n else 1.0
    return 100*lo, 100*hi

if __name__ == '__main__':
    d = pd.read_csv('coding_dataset.csv')
    res = {}
    print(f'Records: {len(d)}  (primary {int((d.document_type=="primary").sum())}, '
          f'review {int((d.document_type=="review").sum())})\n')

    # --- 1. Reviewer agreement, before reconciliation, all 175 records -------
    print('1. INTER-RATER AGREEMENT BEFORE RECONCILIATION (n = 175)')
    print(f'{"domain":32s}{"agree":>8}{"kappa":>8}{"95% CI":>18}')
    res['kappa'] = {}
    for x in DOM:
        k, po, lo, hi = cohen_kappa(d['r1_'+x].values, d['r2_'+x].values)
        res['kappa'][x] = [round(k,3), round(lo,3), round(hi,3), round(100*po,1)]
        print(f'{LAB[x]:32s}{100*po:7.1f}%{k:8.3f}   {lo:.3f} to {hi:.3f}')
    disc = sum(int((d['r1_'+x] != d['r2_'+x]).sum()) for x in DOM)
    res['discordant_record_domain_pairs'] = disc
    print(f'\nDiscordant record-domain pairs reconciled: {disc}\n')

    # --- 2. Domain coverage, consensus, primary studies --------------------
    P = d[d.document_type == 'primary']
    n = len(P)
    print(f'2. DOMAIN COVERAGE, CONSENSUS CODING, PRIMARY STUDIES (n = {n})')
    print(f'{"domain":32s}{"k":>5}{"%":>8}{"95% Wilson CI":>20}')
    res['coverage_primary'] = {}
    for x in DOM:
        k = int(P['cons_'+x].sum()); lo, hi = wilson(k, n)
        res['coverage_primary'][x] = [k, n, round(100*k/n,1), round(lo,1), round(hi,1)]
        print(f'{LAB[x]:32s}{k:5d}{100*k/n:7.1f}%   {lo:5.1f} to {hi:5.1f}')
    both = int(((P.cons_host==1)&(P.cons_commensal==1)).sum())
    lo, hi = wilson(both, n)
    res['host_and_commensal_primary'] = [both, n, round(100*both/n,1), round(lo,1),
```

```
round(hi,1)]
   print(f'{"Host AND commensal":32s}{both:5d}{100*both/n:7.1f}%   {lo:5.1f} to {hi:5.1f}')

   print(f'\n   For comparison, all 175 records:')
   for x in DOM:
      k = int(d['cons_'+x].sum()); lo, hi = wilson(k, len(d))
      print(f'   {LAB[x]:29s}{k:5d}{100*k/len(d):7.1f}%   {lo:5.1f} to {hi:5.1f}')

   # --- 3. Cochran Q and pairwise McNemar, consensus, primary studies ------
   X = np.column_stack([P['cons_'+x].values for x in DOM])
   Q, df, p = cochran_q(X)
   res['cochran_Q'] = [round(Q,2), df, float(p)]
   print(f'\n3. COCHRAN Q (consensus, primary studies)')
   print(f'   Q = {Q:.2f}, df = {df}, {fmt_p(p)}')

   print('\n   Pairwise McNemar, exact, Holm-adjusted:')
   pairs, raw = [], []
   for i in range(len(DOM)):
      for j in range(i+1, len(DOM)):
         c, pv = mcnemar_exact(P['cons_'+DOM[i]].values, P['cons_'+DOM[j]].values)
         pairs.append((DOM[i], DOM[j], c)); raw.append(pv)
   adj = holm(np.array(raw))
   res['mcnemar'] = []
   for (a, b, c), pr, pa in zip(pairs, raw, adj):
      res['mcnemar'].append({'a': a, 'b': b, 'discordant': list(c),
                        'p_exact': float(pr), 'p_holm': float(pa)})
      star = '  *' if pa < 0.05 else ''
      print(f'   {a:12s} vs {b:12s} discordant {str(c):10s} {fmt_p(pa)}{star}')

   print('\n   The same analysis on all 175 records gives identical test results,')
   Xa = np.column_stack([d['cons_'+x].values for x in DOM])
   Qa, dfa, pa_ = cochran_q(Xa)
   print(f'   because the 20 reviews were negative in every domain: Q = {Qa:.2f},
{fmt_p(pa_)}')

   # --- 4. Full-text validation substudy ----------------------------------
   V = d[(d.validation_sample == 1) & (d.full_text_available == 1)]
   print(f'\n4. FULL-TEXT VALIDATION SUBSTUDY (n = {len(V)} of 40 sampled)')
   print(f'{"domain":32s}{"abstract":>10}{"full
text":>11}{"missed":>8}{"added":>7}{"sensitivity":>14}')
   res['validation'] = {}
   tfn = tfp = 0
   for x in DOM:
      ab = V['cons_'+x].astype(int).values
      ft = V['ft_'+x].astype(float).astype(int).values
      fn = int(((ft==1)&(ab==0)).sum()); fp = int(((ft==0)&(ab==1)).sum())
```

```
        tfn += fn; tfp += fp
        k = int((ft & ab).sum()); nn = int(ft.sum())
        lo, hi = clopper_pearson(k, nn) if nn else (0, 0)
        res['validation'][x] = {'abstract': int(ab.sum()), 'full_text': nn,
                                'false_negative': fn, 'false_positive': fp,
                                'sensitivity': round(100*k/nn,1) if nn else None,
                                'ci': [round(lo,1), round(hi,1)]}
        s = f'{100*k/nn:6.1f}% ({k}/{nn})' if nn else '           n/a'
        print(f'{LAB[x]:32s}{int(ab.sum()):10d}{nn:11d}{fn:8d}{fp:7d}{s:>14}')
    res['validation_totals'] = {'false_negative': tfn, 'false_positive': tfp,
                                'record_domain_pairs': len(V)*len(DOM)}
    print(f'\n   Across {len(V)*len(DOM)} record-domain pairs: {tfn} false negatives, {tfp} false positives.')
    print('   Discordance is reported descriptively; pairs within the same record are not independent,')
    print('   so no pooled hypothesis test is performed.')
    nrev = int((V.document_type == 'review').sum())
    print(f'   Reviews among the {len(V)} full texts: {nrev}; excluding them does not change the sensitivities.')

    json.dump(res, open('analysis_S1_output.json','w'), indent=1)
    print('\nWritten: analysis_S1_output.json')
```

Output


```
{
 "kappa": {
  "antifungal": [
   0.75,
   0.65,
   0.85,
   88.0
  ],
  "formulation": [
   0.863,
   0.784,
   0.941,
   93.7
  ],
  "biofilm": [
   0.956,
   0.896,
   1.0,
   98.9
  ],
  "host": [
```

0.8,
0.664,
0.935,
95.4
],
"commensal": [
1.0,
1.0,
1.0,
100.0
]
},
"discordant_record_domain_pairs": 42,
"coverage_primary": {
"antifungal": [
64,
155,
41.3,
33.8,
49.2
],
"formulation": [
59,
155,
38.1,
30.8,
45.9
],
"biofilm": [
27,
155,
17.4,
12.3,
24.2
],
"host": [
25,
155,
16.1,
11.2,
22.7
],
"commensal": [
8,
155,
5.2,

2.6,
9.9
]
},
"host_and_commensal_primary": [
3,
155,
1.9,
0.7,
5.5
],
"cochran_Q": [
79.49,
4,
2.236079439125404e-16
],
"mcnemar": [
{
"a": "antifungal",
"b": "formulation",
"discordant": [
51,
46
],
"p_exact": 0.684858777214404,
"p_holm": 1.0
},
{
"a": "antifungal",
"b": "biofilm",
"discordant": [
48,
11
],
"p_exact": 1.2416282261784595e-06,
"p_holm": 8.691397583249216e-06
},
{
"a": "antifungal",
"b": "host",
"discordant": [
51,
12
],
"p_exact": 7.459722282164659e-07,
"p_holm": 5.967777825731727e-06

},
{
"a": "antifungal",
"b": "commensal",
"discordant": [
60,
4
],
"p_exact": 7.363044635805283e-14,
"p_holm": 7.363044635805283e-13
},
{
"a": "formulation",
"b": "biofilm",
"discordant": [
54,
22
],
"p_exact": 0.0003129786008370502,
"p_holm": 0.001564893004185251
},
{
"a": "formulation",
"b": "host",
"discordant": [
50,
16
],
"p_exact": 3.328235017032062e-05,
"p_holm": 0.00019969410102192373
},
{
"a": "formulation",
"b": "commensal",
"discordant": [
56,
5
],
"p_exact": 5.645556938604912e-12,
"p_holm": 5.081001244744421e-11
},
{
"a": "biofilm",
"b": "host",
"discordant": [
19,

17
],
"p_exact": 0.8679394004284404,
"p_holm": 1.0
},
{
"a": "biofilm",
"b": "commensal",
"discordant": [
24,
5
],
"p_exact": 0.0005461126565933228,
"p_holm": 0.002184450626373291
},
{
"a": "host",
"b": "commensal",
"discordant": [
22,
5
],
"p_exact": 0.0015137195587158203,
"p_holm": 0.004541158676147461
}
],
"validation": {
"antifungal": {
"abstract": 14,
"full_text": 27,
"false_negative": 13,
"false_positive": 0,
"sensitivity": 51.9,
"ci": [
31.9,
71.3
]
},
"formulation": {
"abstract": 9,
"full_text": 12,
"false_negative": 3,
"false_positive": 0,
"sensitivity": 75.0,
"ci": [
42.8,

94.5
]
},
"biofilm": {
"abstract": 10,
"full_text": 12,
"false_negative": 2,
"false_positive": 0,
"sensitivity": 83.3,
"ci": [
51.6,
97.9
]
},
"host": {
"abstract": 5,
"full_text": 10,
"false_negative": 5,
"false_positive": 0,
"sensitivity": 50.0,
"ci": [
18.7,
81.3
]
},
"commensal": {
"abstract": 7,
"full_text": 7,
"false_negative": 0,
"false_positive": 0,
"sensitivity": 100.0,
"ci": [
59.0,
100.0
]
}
},
"validation_totals": {
"false_negative": 23,
"false_positive": 0,
"record_domain_pairs": 145
}
}

Cross-corpus sensitivity analysis

The routine that produced the figures reported in Section 3.4.1 of the main text is provided as sensitivity_S1.py, together with its printed output as sensitivity_S1_output.txt. It applies the term dictionary of S1.7 without modification to three independently retrieved corpora and reports, for each, the prevalence of the five domains with Wilson intervals, Cochran's Q, and the resulting ordering. The prespecified outcome defined in S1.10 is the ordering, not the individual proportions.

Files added at revision. coding_dataset_extended.csv contains the 321 records of the extended corpus in the format of coding_dataset.csv with three additional columns (corpus_block, original or extension; screen_id, the identifier used in the screening workbooks; wos_ut, the Web of Science accession number of the added records). analysis_S1_extended.py applies the analysis of analysis_S1.py to the original subset, to the extension block, and to the extended corpus, and writes analysis_S1_extended_output.json and analysis_S1_extended_output.txt. screening_sweep_S1.py reproduces the 4,814-record denominator and the 175-record filter, runs the expanded sweep, and draws the dual-screening sample with the retained seed. The completed workbooks of the dual screening (step 1), the reconciliation and third batch (step 2), and the domain coding of the added records (steps 3 and 4) are supplied as received from the evaluators.

Running the script requires the raw exports listed below, which are provided with it. Filenames are those expected by the script.

File	Content
wos_population_block_0.xlsx to wos_population_block_5.xlsx	Web of Science population block as exported, six batches, 5,385 records; the plant-derived subset is derived by the script
scopus_S3_plant_derived.csv	Scopus export, population block AND plant-derived block, 316 records, with abstracts and both keyword fields
pubmed_S3_plant_derived.csv	PubMed export, population block AND plant-derived block, 251 records; contains titles only, retained to document why the corpus is not comparable
sensitivity_S1.py	Analysis routine
sensitivity_S1_output.txt	Printed output of the routine as executed

The Web of Science plant-derived subset is not supplied as a separate file, because it is defined by the term dictionary rather than by a separate search, and the script derives it reproducibly from the population block.
